# Supplementary material for: Ubiquitylation by the GID/CTLH complex regulates the metabolic and innate immune response of macrophages to infection by Mycobacterium tuberculosis
Source: PLoS Pathog. 2026 Sep 18;22(9):e1014196. doi: 10.1371/journal.ppat.1014196 (PMC13614649; doi:10.1371/journal.ppat.1014196)
Supplement: Supplementary file 1 [file ppat.1014196.s018.pdf]

S1_Raw_Images

Fig 5A

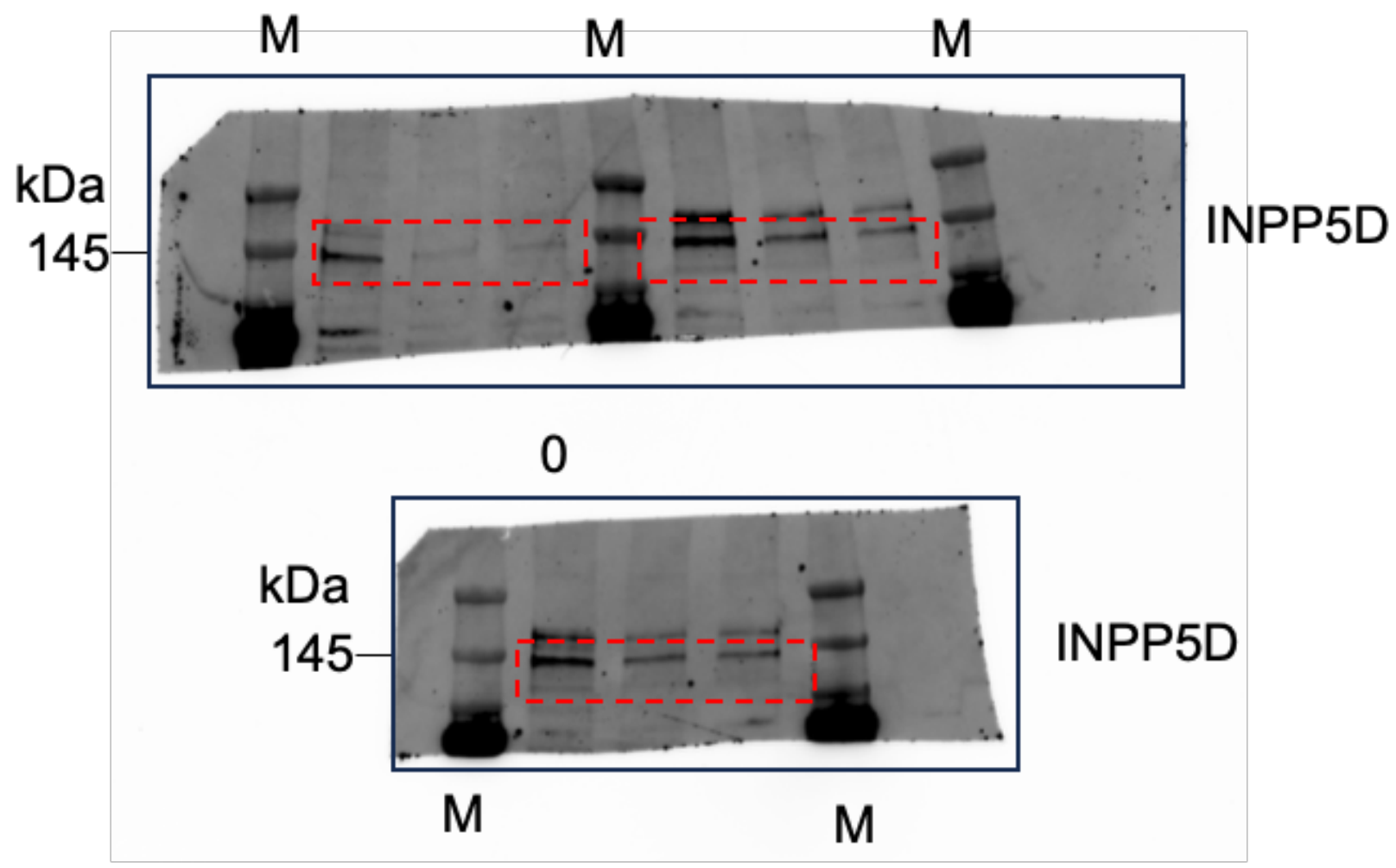

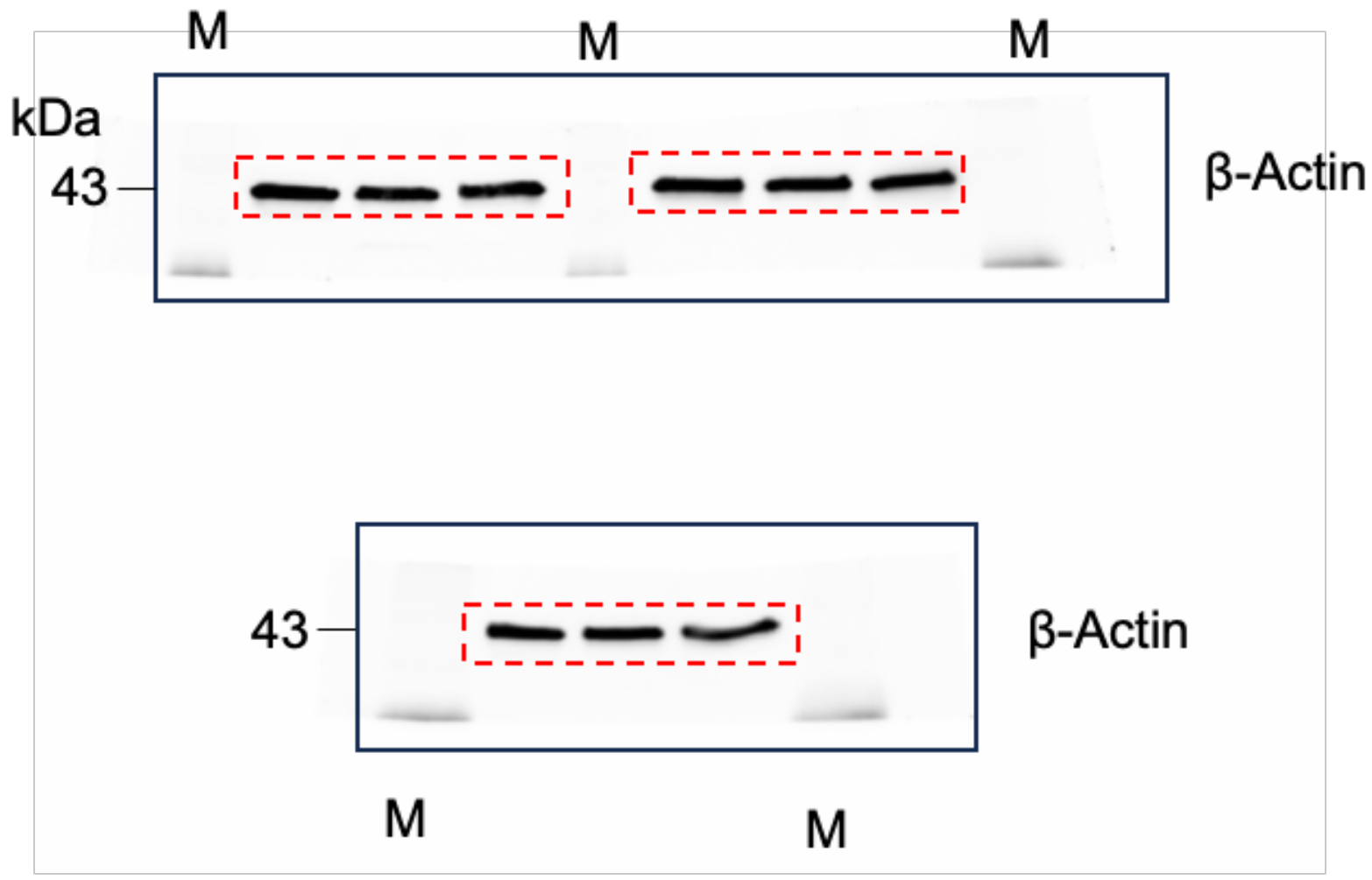

Labels for each target

- Top left to right: Scramble, GID8$^{-/-}$ g1 as in Fig 5A
- Bottom left to right: MAEA$^{-/-}$ g2 as in Fig 5A
- M: marker

Fig 5B

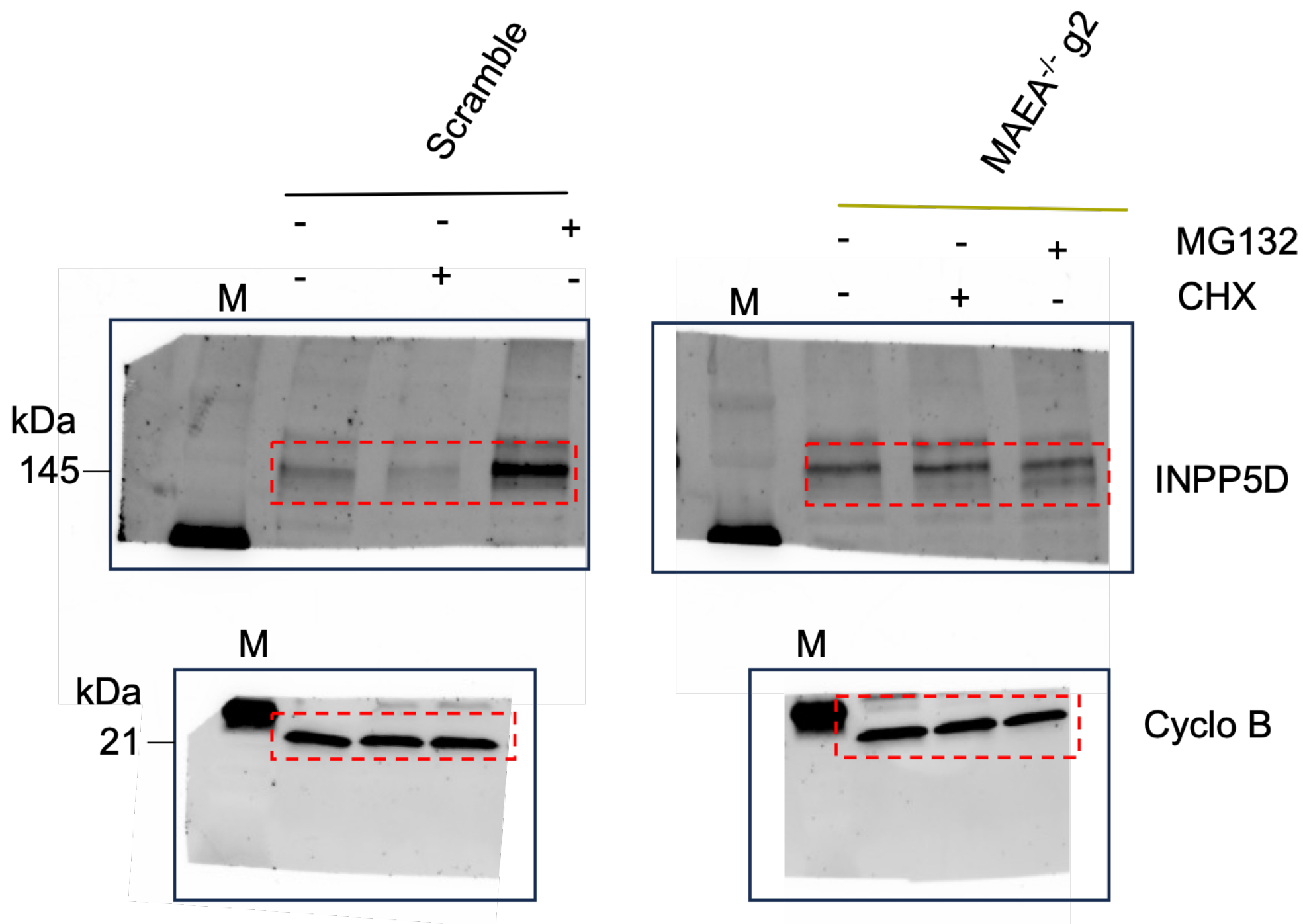

Fig 5C

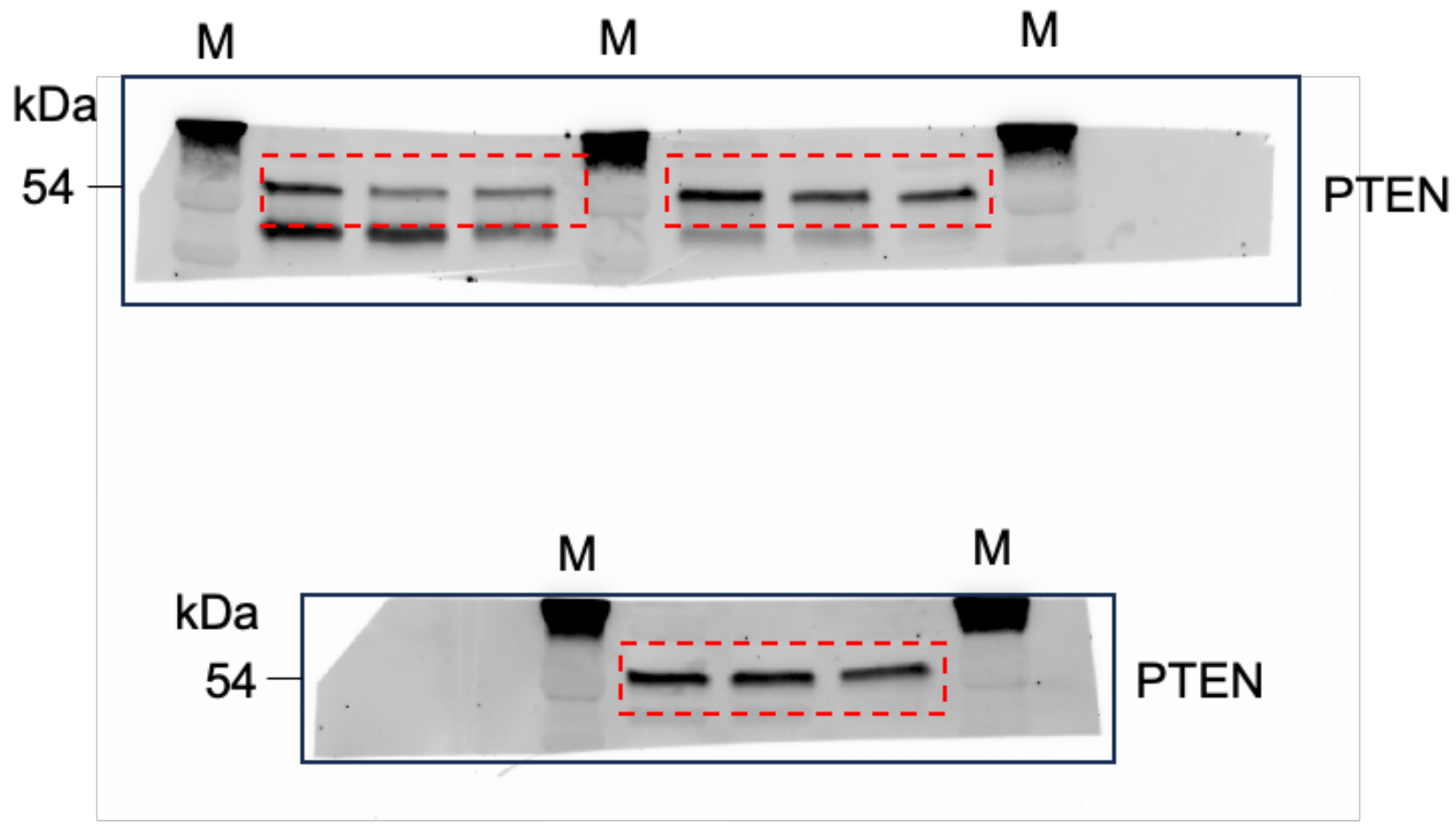

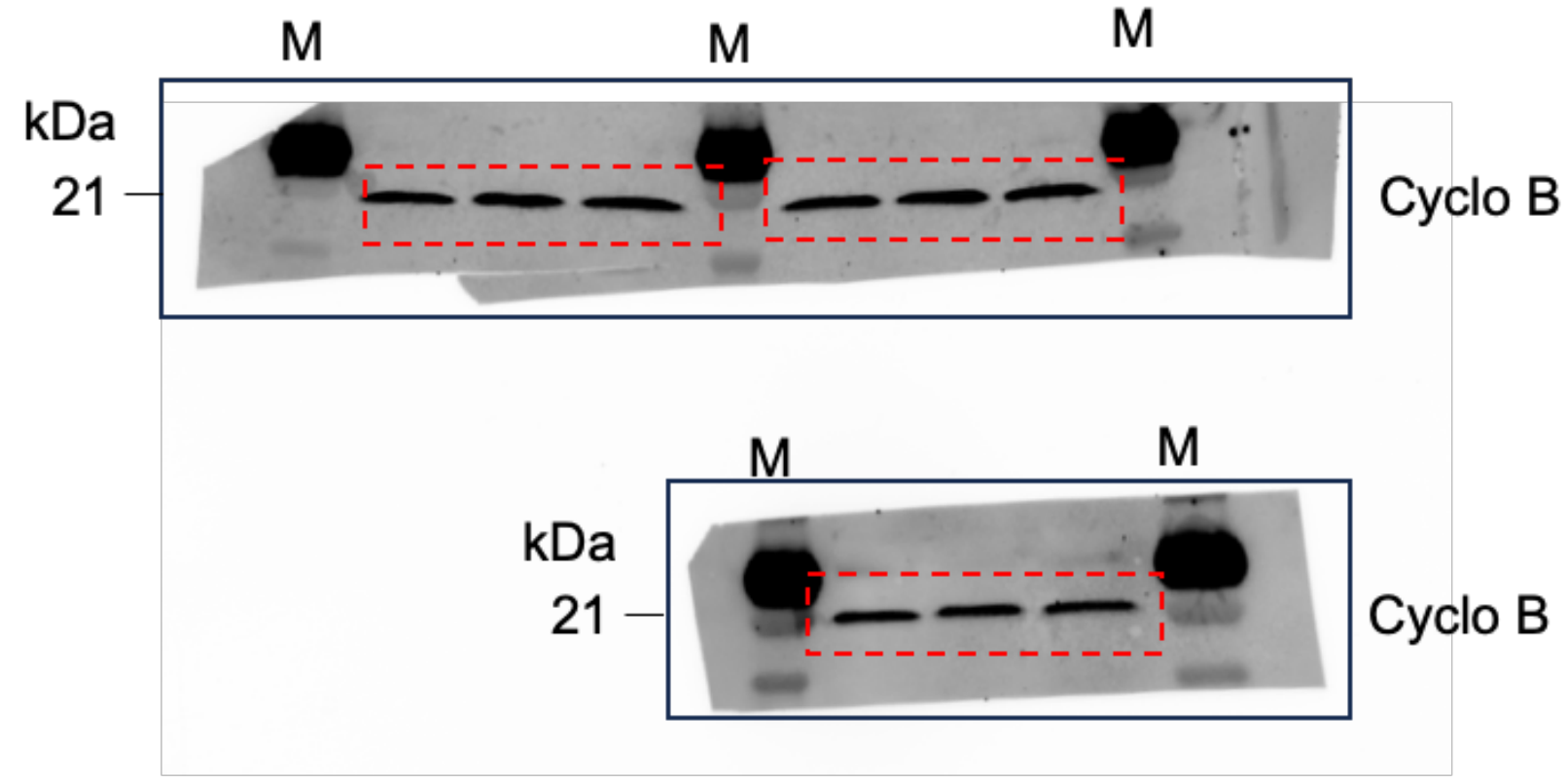

Labels for each target

- Top left to right: Scramble, $GID8^{-/-}$ g1 as in Fig 5C
- Bottom left to right: $MAEA^{-/-}$ g2 as in Fig 5C
- M: marker

Fig 6A

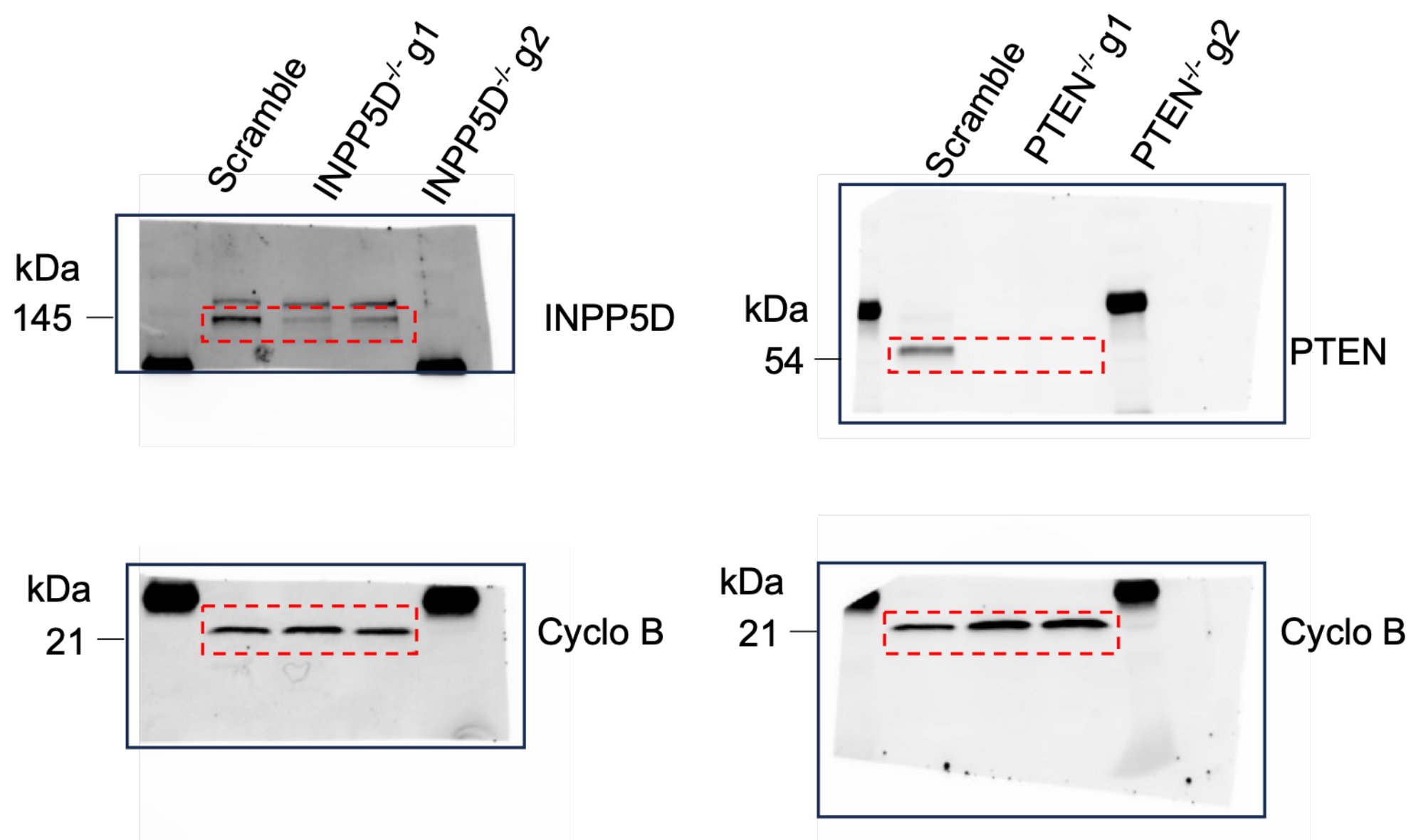
Scramble
INPP5D-/- g1
INPP5D-/- g2
kDa
145
INPP5D
Scramble
PTEN-/- g1
PTEN-/- g2
kDa
54
PTEN
kDa
21
Cyclo B
kDa
21
Cyclo B

Fig 7A

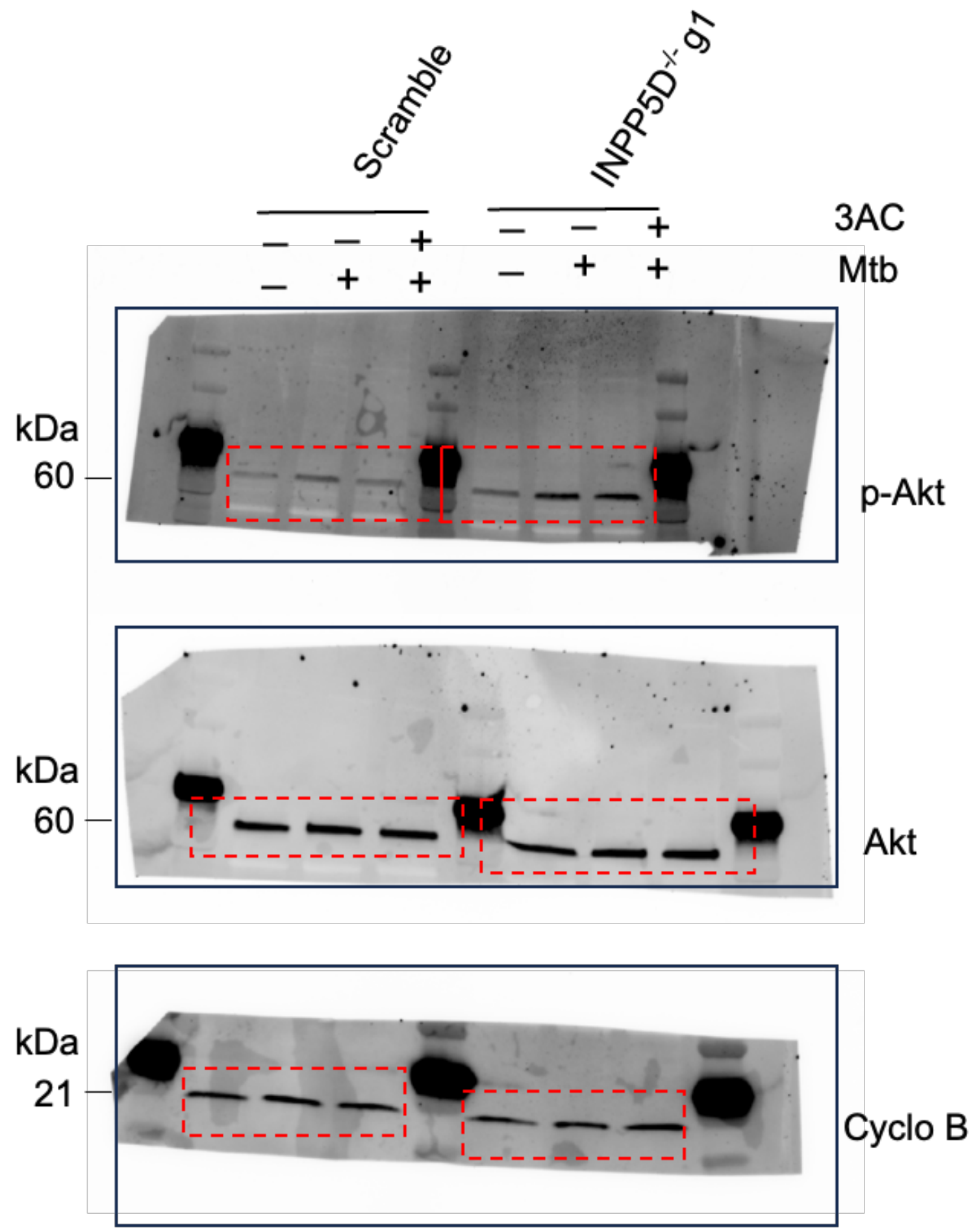

Note: the 3AC data, including the corresponding blot panels in this figure, have now been removed from the main manuscript.

S5A Fig

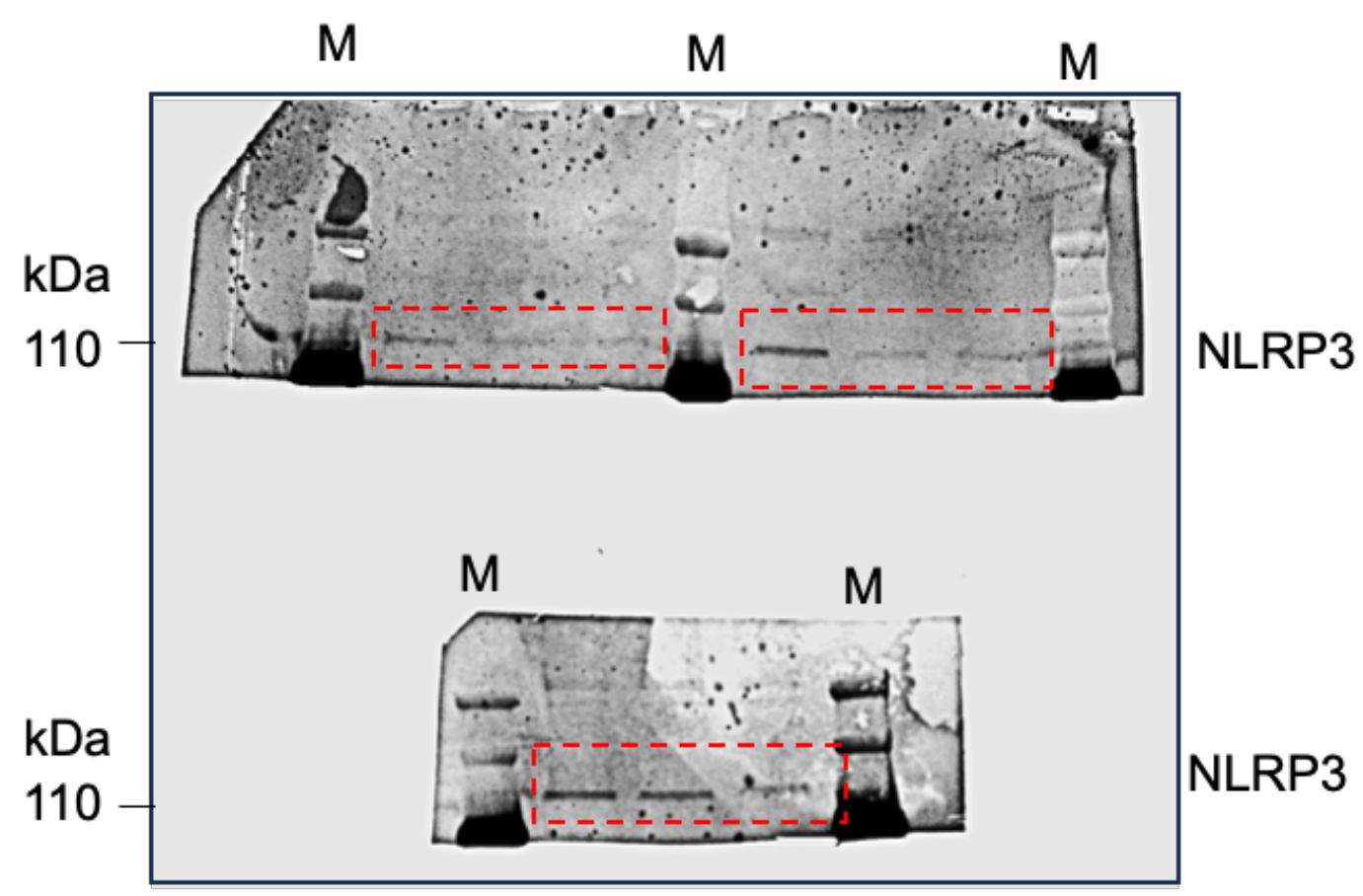

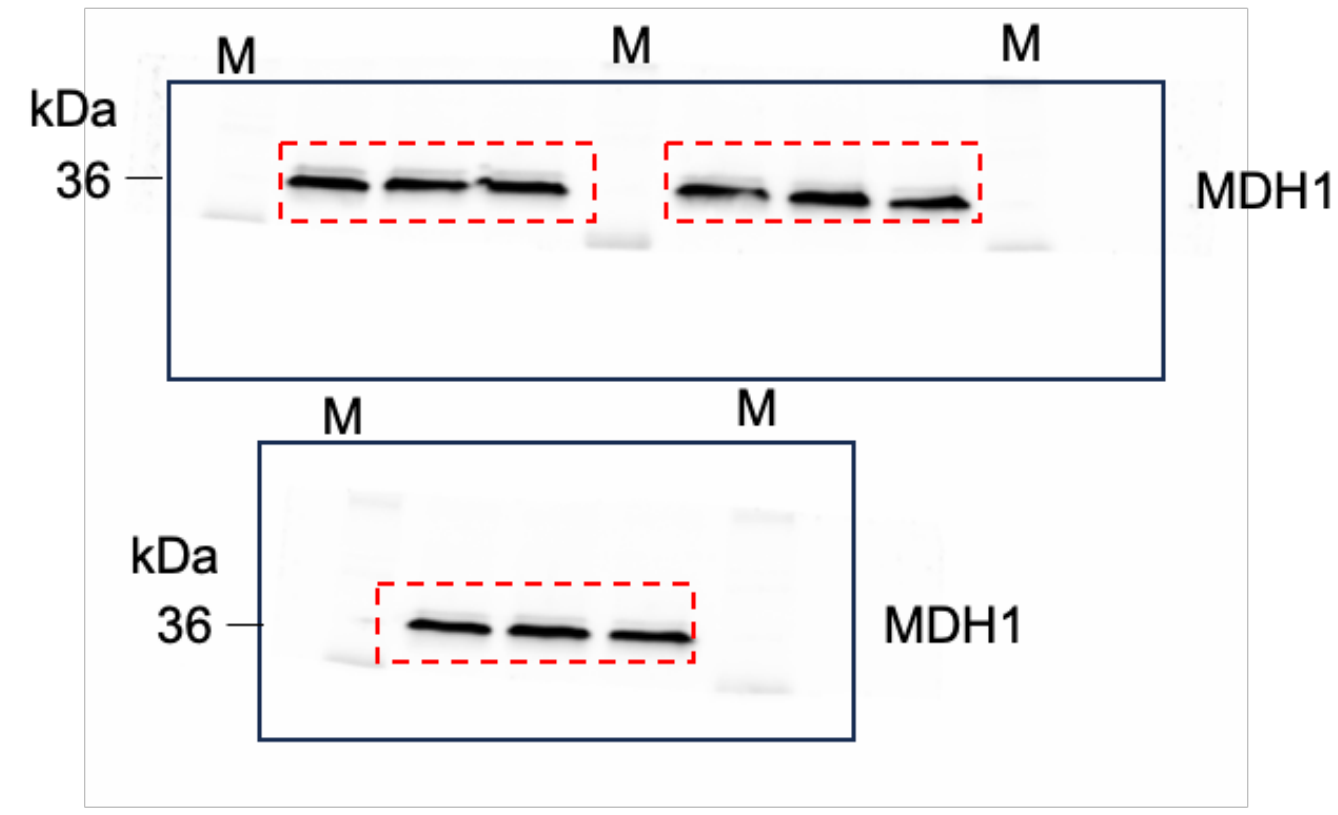

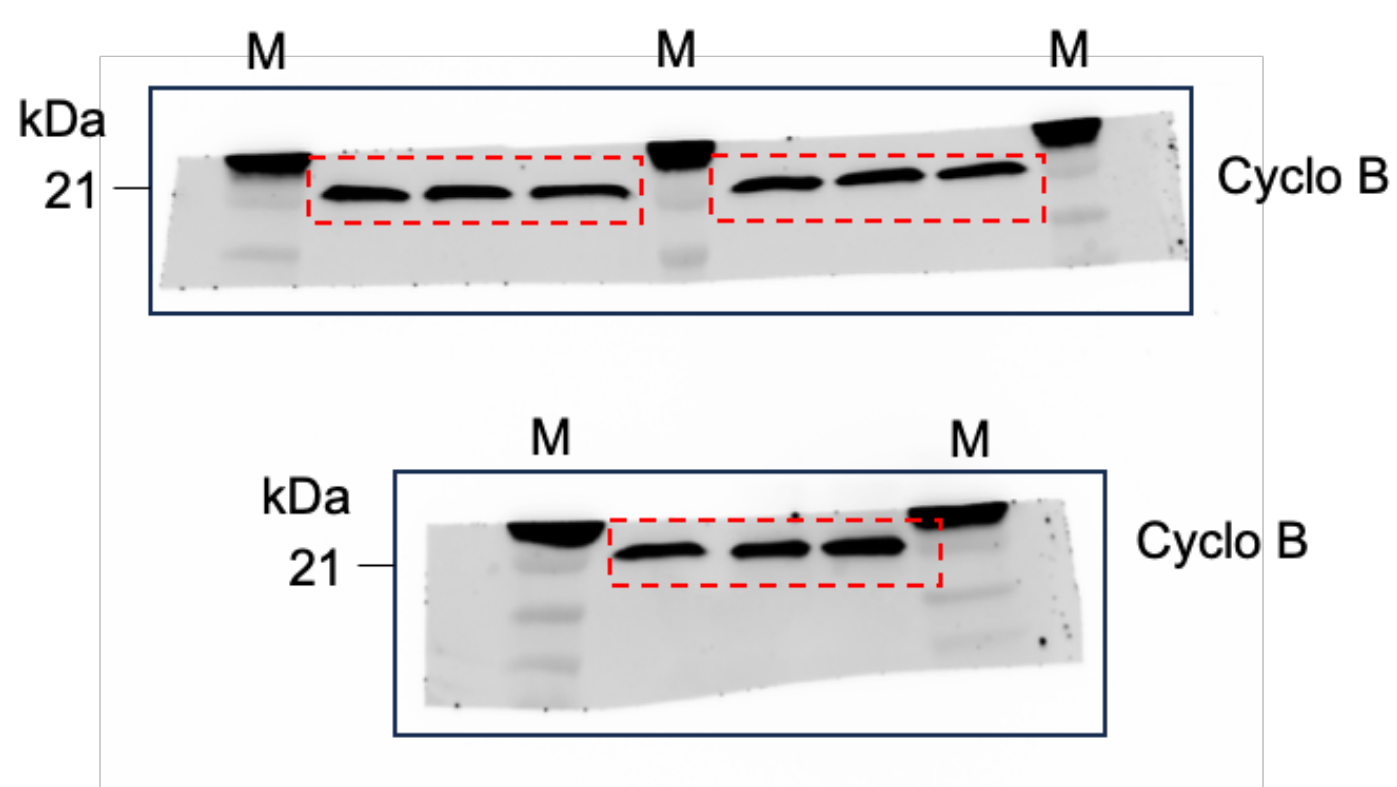

Labels for each target

- NLRP3 top left to right: Scramble, GID8$^{-/-}$ g1 as in S5A Fig
- NLRP3 bottom left to right: MAEA$^{-/-}$ g2 as in S5A Fig
- MDH1 top left to right : Scramble, GID8$^{-/-}$ g1 as in S5A Fig
- MDH1 bottom left to right : MAEA$^{-/-}$ g2 as in S5A Fig
- Cyclo B top left to right : Scramble, GID8$^{-/-}$ g1 as in S5A Fig
- Cyclo B bottom left to right: MAEA$^{-/-}$ g2 as in S5A Fig
- M: marker

S5B Fig

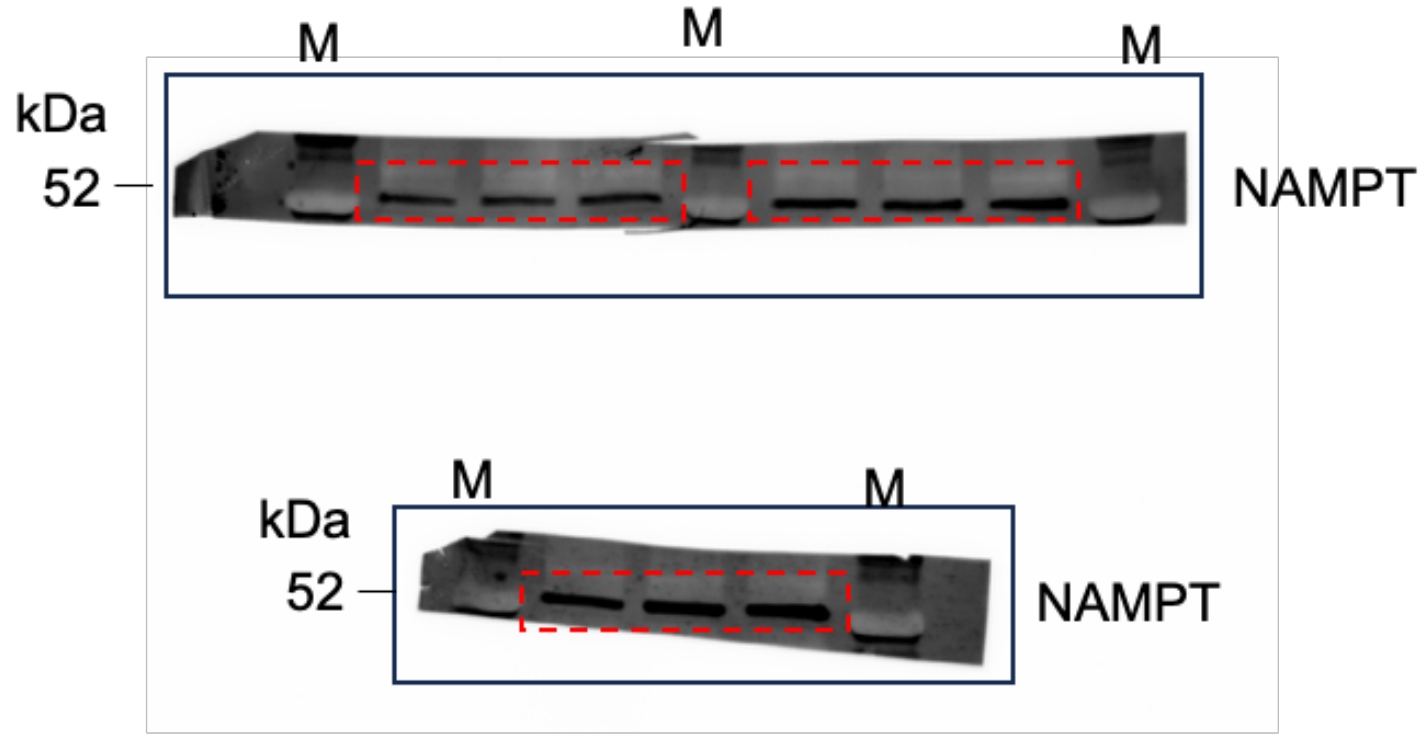

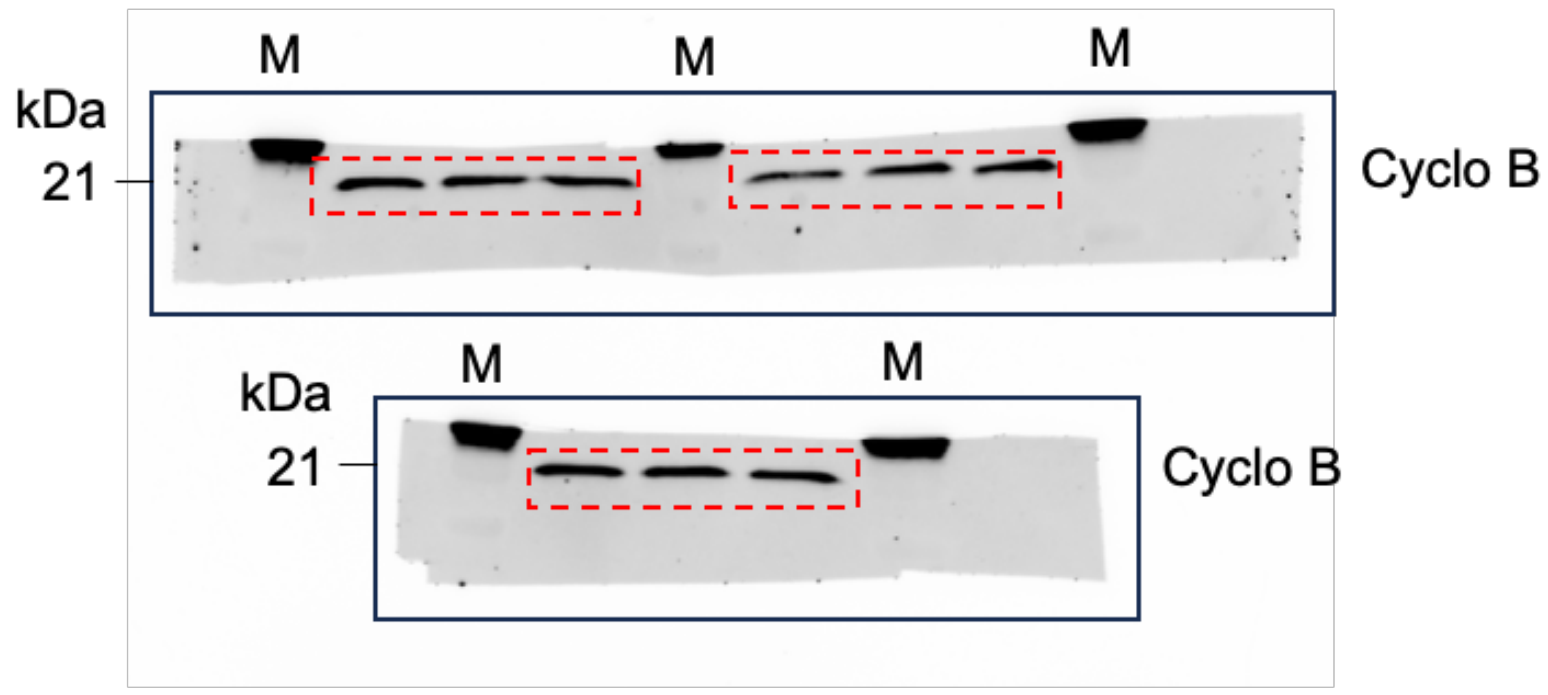

Labels for each target

- NAMPT top left to right: Scramble, GID8$^{-/-}$ g1 as in S5B Fig
- NAMPT bottom left to right: MAEA$^{-/-}$ g2 as in S5B Fig
- Cyclo B top left to right : Scramble, GID8$^{-/-}$ g1 as in S5B Fig
- Cyclo B bottom left to right : MAEA$^{-/-}$ g2 as in S5B Fig
- M: marker

S5C Fig

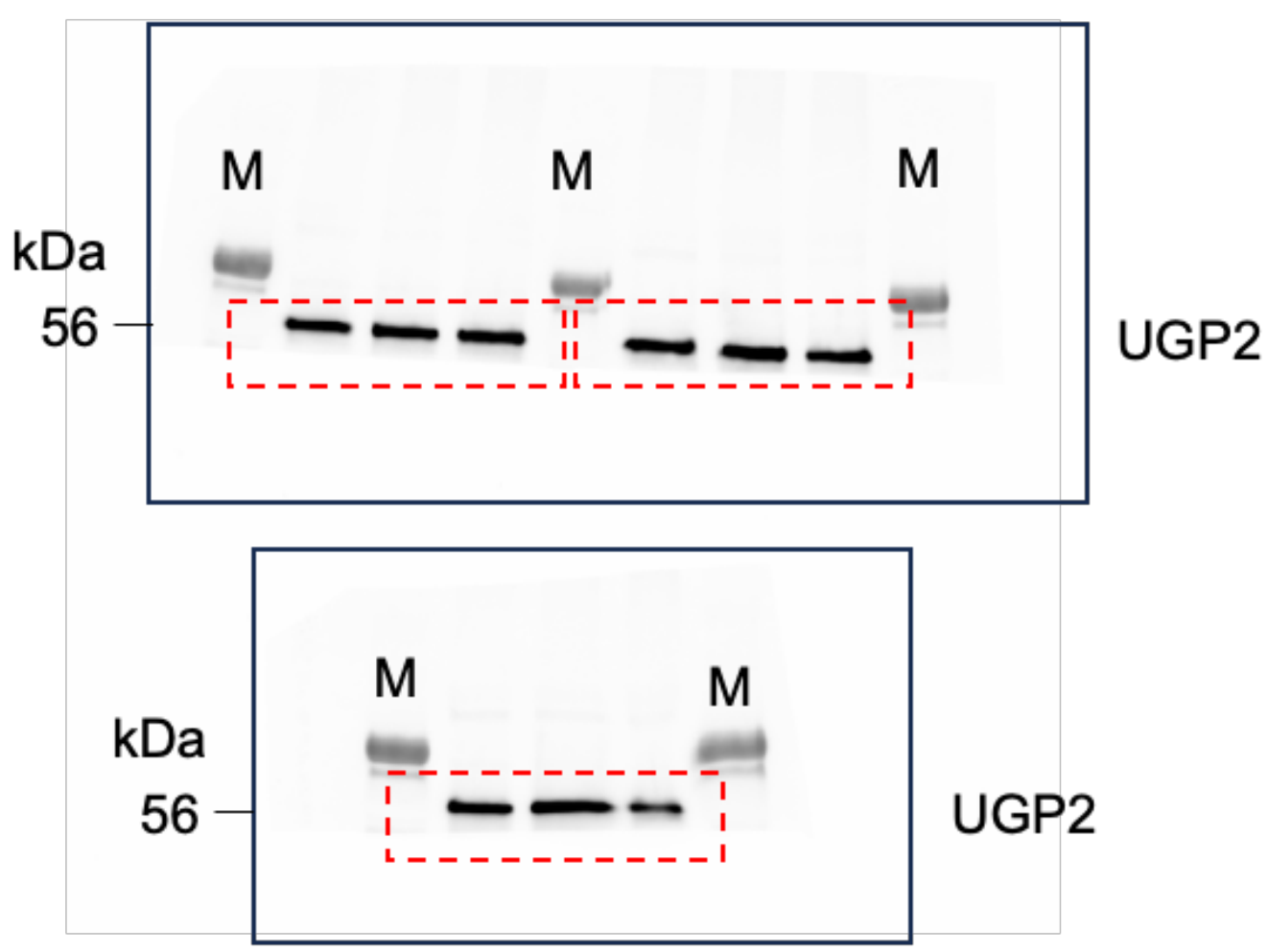

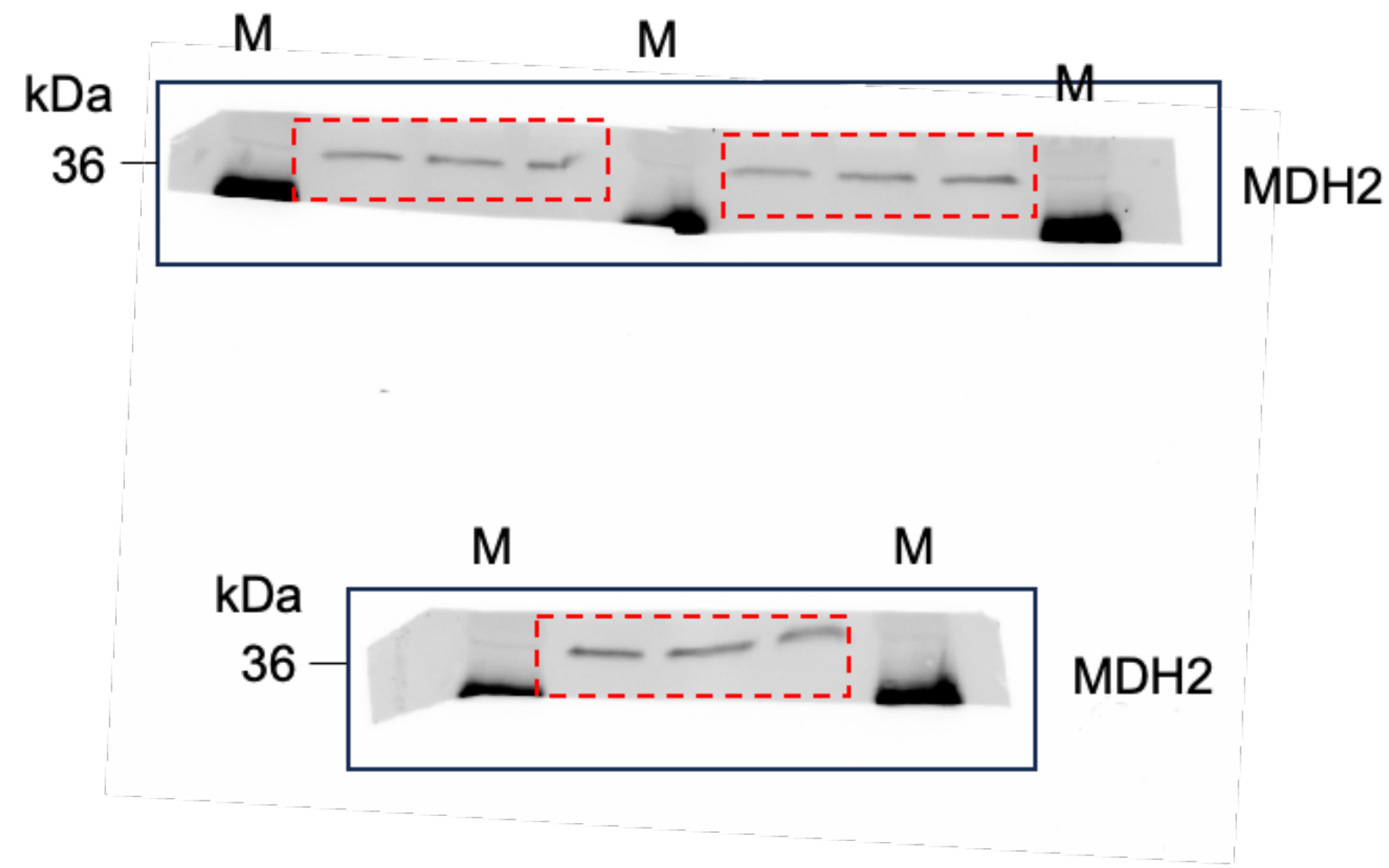

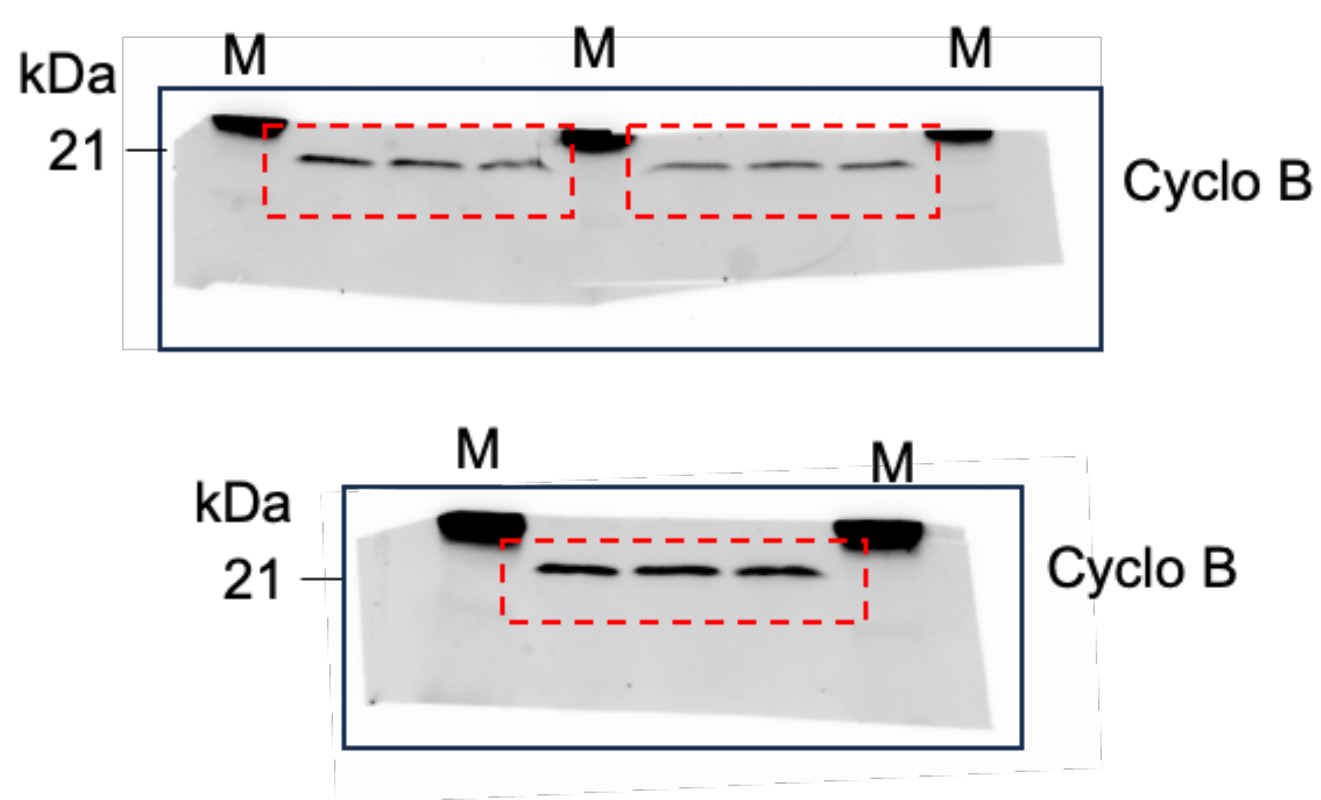

Labels for each target

- UGP2 top left to right: Scramble, GID8$^{-/-}$ g1 as in S5C Fig
- UGP2 bottom left to right: MAEA$^{-/-}$ g2 as in S5C Fig
- MDH2 top left to right : Scramble, GID8$^{-/-}$ g1 as in S5C Fig
- MDH2 bottom left to right : MAEA$^{-/-}$ g2 as in S5C Fig
- Cyclo B top left to right : Scramble, GID8$^{-/-}$ g1 as in S5C Fig
- Cyclo B bottom left to right: MAEA$^{-/-}$ g2 as in S5C Fig
- M: marker

S5D Fig

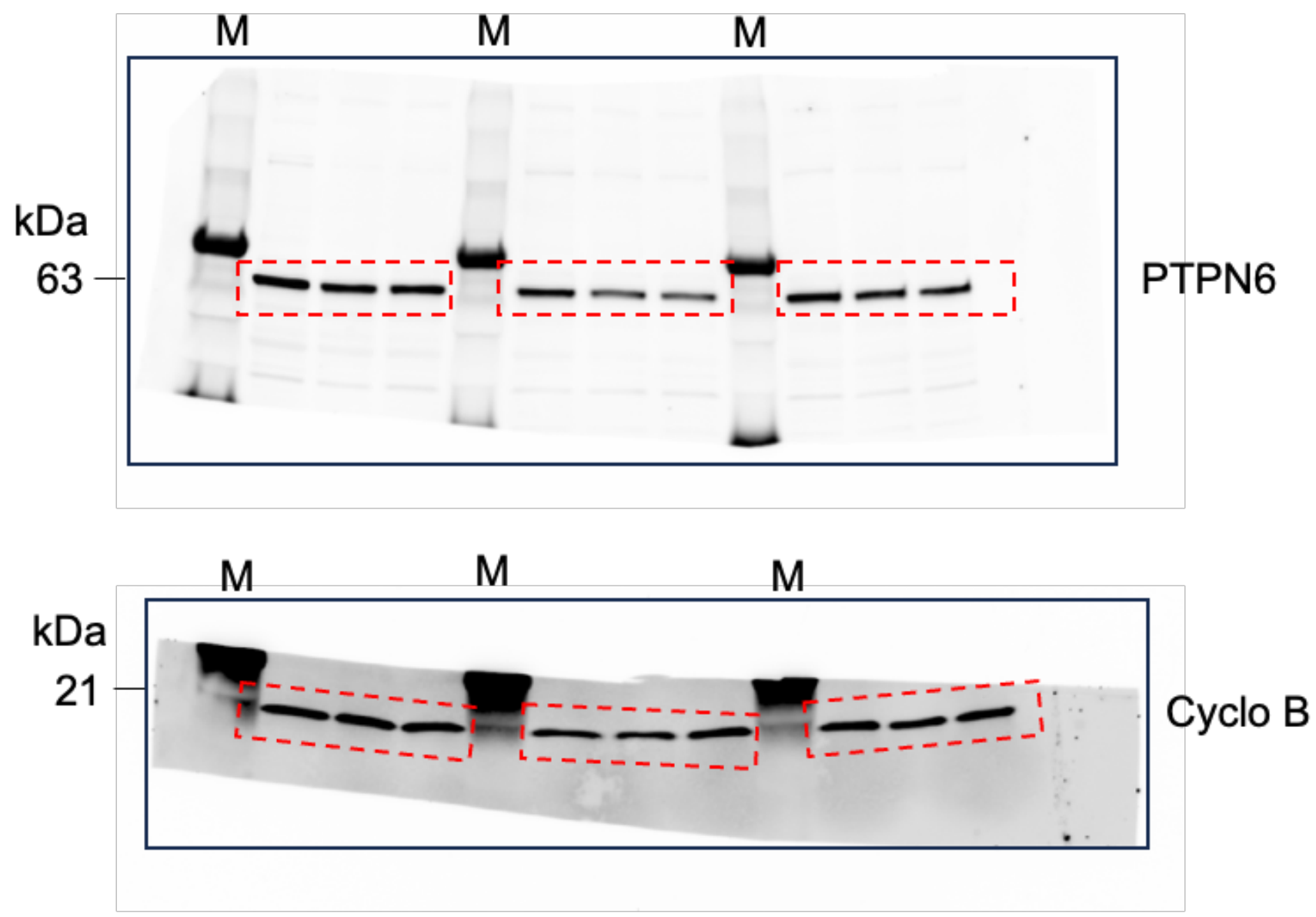

Labels for each target

- PTPN6: left to right: Scramble, GID8$^{-/-}$ g1, MAEA$^{-/-}$ g2 as in S5D Fig
- Cyclo B: left to right: Scramble, GID8$^{-/-}$ g1, MAEA$^{-/-}$ g2 as in S5D Fig
- M: marker

S6A Fig

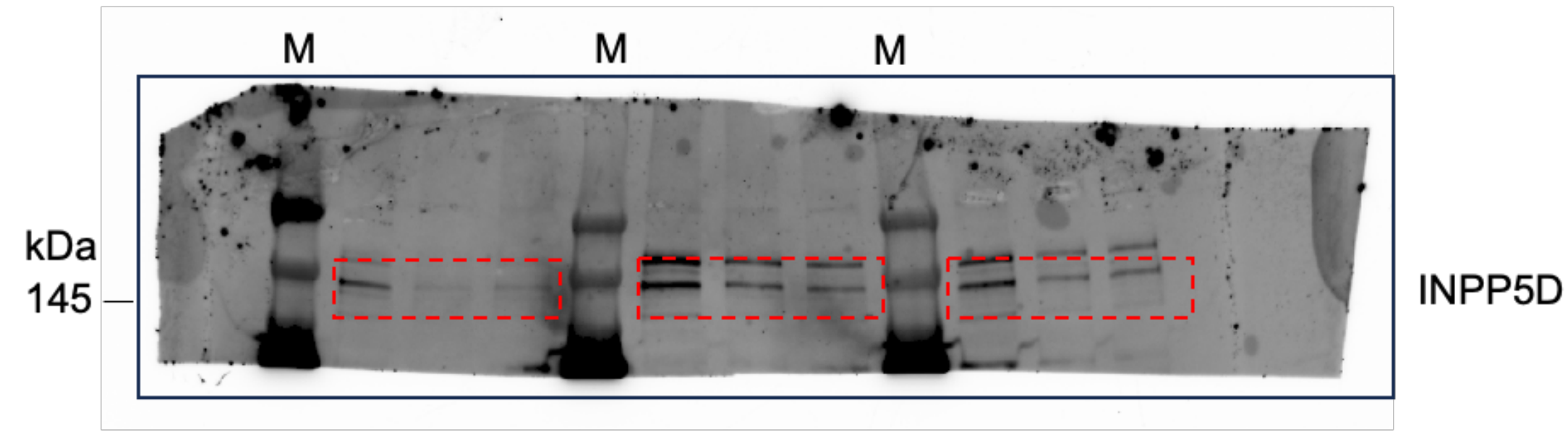

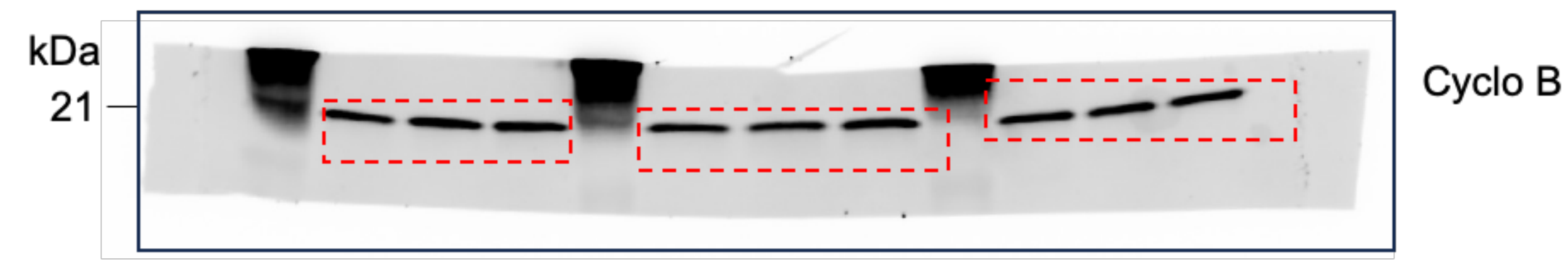

Labels for each target

- INPP5D: left to right: Scramble, GID8$^{-/-}$ g1, MAEA$^{-/-}$ g2 as in S6A Fig
- Cyclo B: left to right: Scramble, GID8$^{-/-}$ g1, MAEA$^{-/-}$ g2 as S6A Fig
- M: marker

S6B Fig

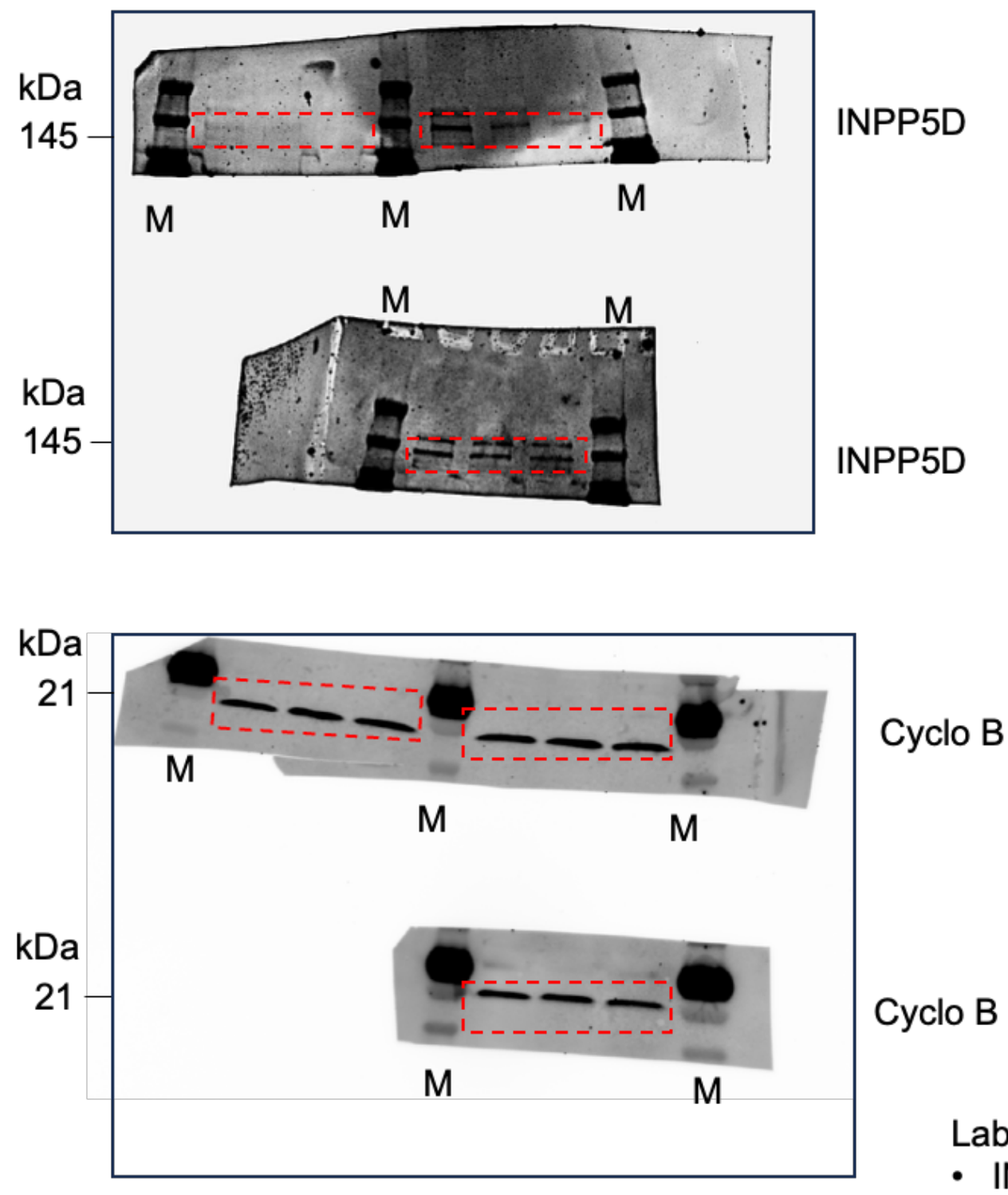

Labels for each target

- INPP5D top left to right: Scramble, GID8$^{-/-}$ g1 as in S6A Fig
- INPP5D bottom left to right: MAEA$^{-/-}$ g2 as in S6A Fig
- Cyclo B top left to right : Scramble, GID8$^{-/-}$ g1 as in S6A Fig
- Cyclo B bottom left to right : MAEA$^{-/-}$ g2 as in S6A Fig
- M: marker

S6C Fig

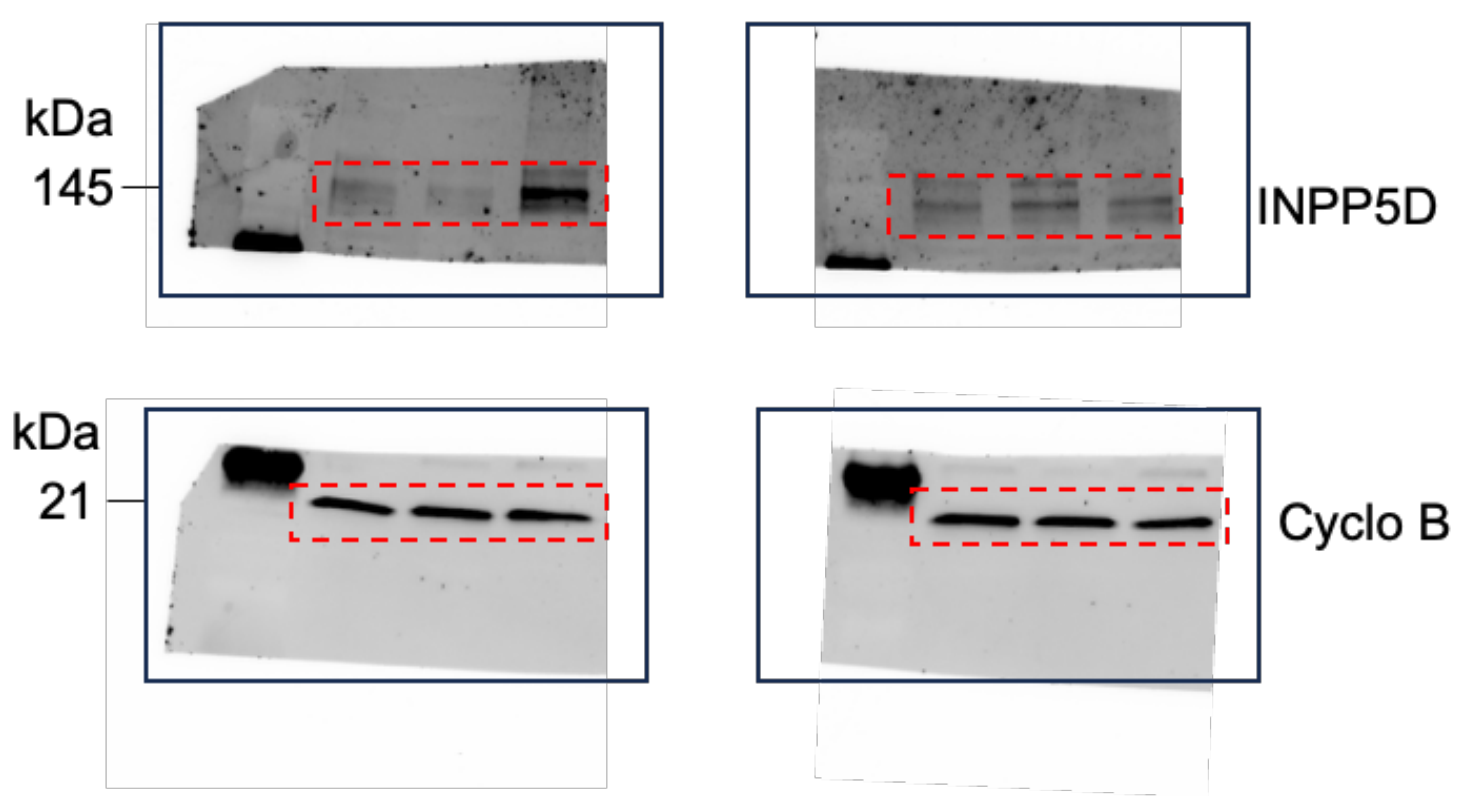

Labels are the same as in raw image 5B

S6D Fig

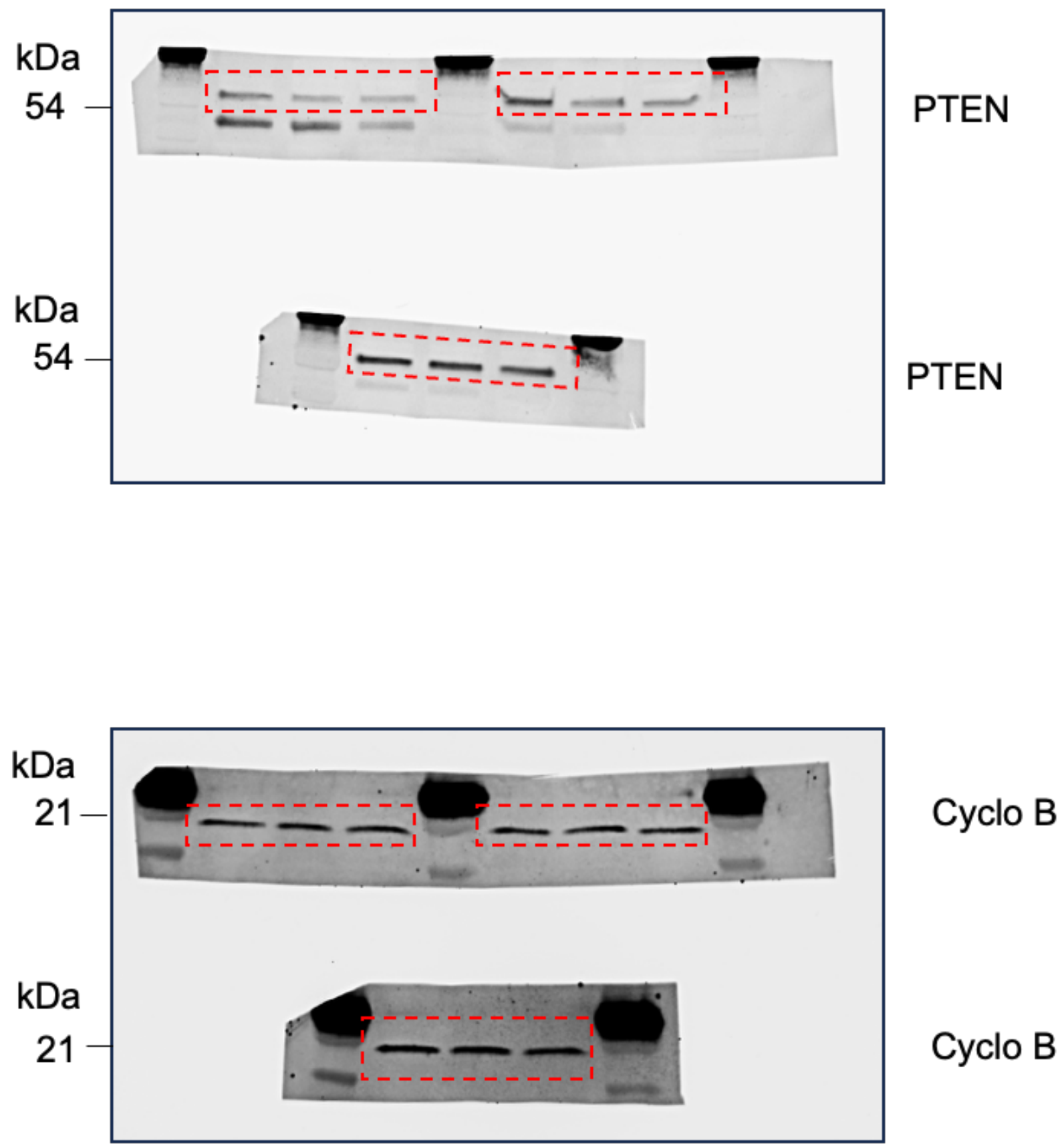

Labels are the same as in raw image 5C

S7A Fig

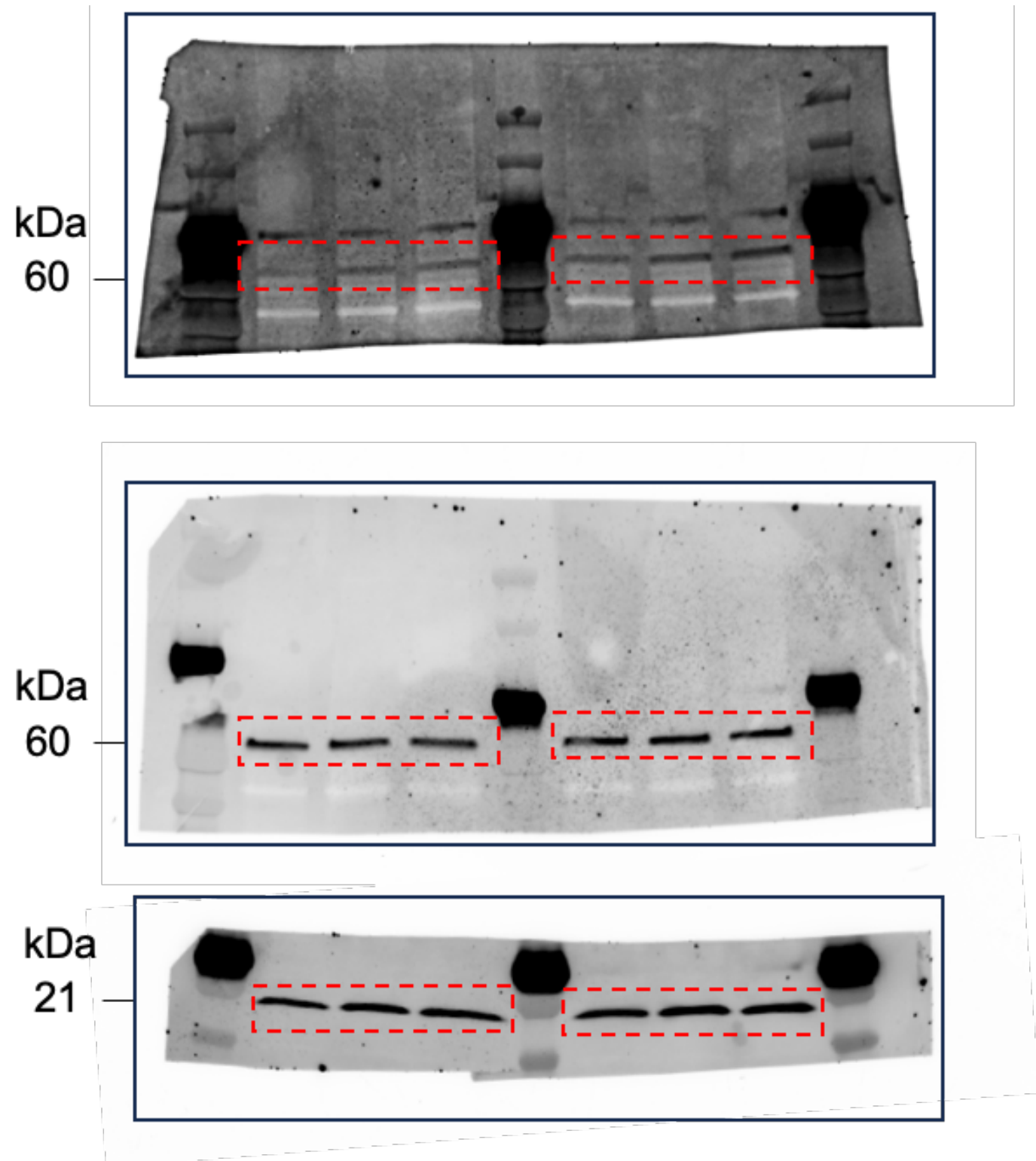

Labels are the same as in raw image 7A

Note: the 3AC data, including the corresponding blot panels in this figure, have now been removed from the main manuscript.
