# Supplementary material for: Ubiquitylation by the GID/CTLH complex regulates the metabolic and innate immune response of macrophages to infection by Mycobacterium tuberculosis
Source: PLoS Pathog. 2026 Sep 18;22(9):e1014196. doi: 10.1371/journal.ppat.1014196 (PMC13614649; doi:10.1371/journal.ppat.1014196)
Supplement: Supplementary file 2 [file ppat.1014196.s020.docx]

**Reviewers' Comments:**

Reviewer's Responses to Questions

**Part I - Summary**
Please use this section to discuss strengths/weaknesses of study, novelty/significance, general execution and scholarship.

**Reviewer #1:** In this manuscript, the authors explore how the CTLH complex contributes to reshaping the proteome during Mtb infection in macrophages. This work builds directly upon previous work in which the authors identified CTLH in a screen to find host factors that impair control of Mtb intracellular infection. Using comparative proteomics, they identify significant metabolic rewiring of metabolism in cells lacking CTLH components GID8 or MAEA, including increased abundance of proteins in oxidative phosphorylation pathways, increased abundance of lipid and fatty acid catabolic pathway proteins, and increase in abundance of proteins associated with reactive oxygen species. Proteins associated with innate immune functions are decreased in abundance, suggesting dampened inflammation. As CTLH has E3 ligase activity, the authors then perform comparative analysis of ubiquitinylated proteins to identify candidate substrates that could mediate the observed effects. Among candidate substrates, they focus on PTEN and INPP5D, finding that loss of INPP5D enhances pro-inflammatory gene expression- of note, this is in contrast to the immune dampening effect of the CTLH complex. The use of comparative proteomic approaches to understand the contributions of individual host factors to the macrophage response to Mtb infection is both unusual and valuable, and the use of two distinct CTLH subunits in the analysis is a nice validation of key findings. On balance, the work is a meaningful contribution to our understanding of how macrophages respond to Mtb, specifically the role of the CTLH complex in regulation of the immune and metabolic states of the cells.

**Reviewer #2:** Global protein abundance and diGly proteomic analyses were performed in scramble control, GID8, and MAEA deficient Hoxb8 BMDMs following Mtb infection. This led the investigators to test the hypothesis that the loss of GID/CTLH activity regulates metabolic and innate immune pathways. The authors focused on two candidate GID/CTLH substrates: the inhibitor phosphatases INPP5D and PTEN. Cycloheximide chase assays and Mtb growth assays in inhibitor phosphatase mutant cells revealed that the phosphatases were degraded by proteosomes, confirmed that the phosphatases are candidate GID/CTLH substrates, and revealed that Mtb growth was restricted in phosphatase-deficient cells by CFU but not luciferase assays. Mtb-infected GID/CTLH-deficient displayed downregulation of pro-inflammatory genes. The manuscript’s strengths are that it provides important insights into host ubiquitin ligases and their substrate phosphatases that control Mtb growth in macrophages and inflammatory responses. Furthermore, detailed follow up mechanistic studies were performed on several of the hits from the global ubiquitin profiling assays. The authors used both pharmacologic and genetic strategies to perturb host phosphatases. A weakness is that the experiments are performed in engineered HoxB8 BMDMs, although these have been shown to display transcriptional responses to Mtb that are similar to unmodified primary BMDMs. Additional weaknesses are described below.

**Reviewer #3:** The study by Simwela et al seeks to understand how ubiquitiylation regulates macrophage-intrinsic responses to Mycobacterium tuberculosis (Mtb). It follows up on the findings of an elegant genome-wide CRISPR screen that identified components of the GID/CTLH E3 ligase complex as important in regulating metabolism and restricting Mtb growth in macrophages. They carry out several quantitative MS experiments to profile changes in these cells and then follow-up with biochemical analyses of several selected proteins and inhibitors.

There are several strong aspects to the current study including in-depth proteomic analysis of both total protein and di-Gly modifications in GID-deficient cells with careful bioinformatic pathway enrichment analysis to identify processes that may be regulated by GID. The total protein analysis is rigorous and they were able to profile a large fraction of the expressed proteome with rigorous quantification. Notably, their pathway analysis demonstrates a convincing enrichment of core metabolic processes involved in ATP generation in KGID/CTL KO macrophages. Their CHX-chase experiments are also convincing, and demonstrate stabilization of SHIP1 and PTEN.

However there are several aspects of this study that are not as well developed. In some cases the proteomic analyses are missing important analyses that introduce confounding variables and the PTM data hard to interpret. In addition, most of the data are descriptive analyses of proteome or transcriptome changes in mutant cells or inhibitor-treated cells, with few mechanistic experiments included. When there are specific hypotheses tested many of the phenotypes are small, such as the changes in Mtb growth in mutant macrophages. Finally, there are methodologic concerns about some of the experiments. The above concerns are detailed below:

We thank the reviewers for taking the time to review our manuscript and for their valuable insights and summary of our work. We provide point-by-point responses to the issues and concerns raised in the sections below.

**Part II – Major Issues: Key Experiments Required for Acceptance**
Please use this section to detail the key new experiments or modifications of existing experiments that should be absolutely required to validate study conclusions.

Generally, there should be no more than 3 such required experiments or major modifications for a "Major Revision" recommendation. If more than 3 experiments are necessary to validate the study conclusions, then you are encouraged to recommend "Reject".

**Reviewer #1:** Unclear rationale for use of 3AC- Given that 3AC has pleiotropic effects that are difficult to disentangle, inhibiting both INPP5D and Mtb growth independent of the macrophage, it’s difficult to know how to interpret 3AC data. On balance, the 3AC data may add more confusion than clarity to the key messages of the manuscript, and I would favor removing the 3AC data.

We agree with the reviewer that the 3AC data are challenging and ought not to be over sold, however, we wished to retain it in the study because it provides independent validation of the altered macrophage phenotype achieved by gene deletion, independent of its direct activity on Mtb. 3AC is the most widely used INPP5D inhibitor and has been extensively employed to confirm and characterize the immunological involvement of INPP5D (SHIP1) in macrophages (e.g., PMIDs: 20200281, 38016942), including BMDMs infected with Mtb (PMID: 27681624). We observed target engagement by the inhibitor, including partial rescue of activity in INPP5D KO BMDMs (Fig 6C) and moderately increased Akt phosphorylation in inhibitor-treated cells (Fig 7 and S7A Fig), both of which mirror the genetic knockout condition. We have revised the corresponding results section, and its title, to better clarify the strengths and weakness of the 3AC data (Lines 422-457). If these data were omitted, we suspect that researchers familiar with this pathway would question why we did not use chemical inhibitors.

No validation of the comparative proteomic data: The authors note that GID8 and MAEA proteins are depleted in their respective knockout conditions, but there is no other validation of the comparative proteomics findings. Given the heavy weight placed on pathway analysis of these data in the manuscript, some additional validation of at least a few differentially abundant proteins by Western blotting would increase confidence in those interpretations.

We thank the reviewer for pointing this out. Indeed, GID8 and MAEA proteins were depleted in their respective KO cells and served, in part, as internal validation of our proteomics pipeline. We did not perform additional Western blot analyses to confirm their depletion, as we had already demonstrated the loss of GID8 and MAEA at both protein and transcript levels in our previous study (PMID: 39472457**)**. We have revised this text to include clear reference to our previous study, most specifically the depletion of these proteins (Lines 179-182).

No cell viability assay in the PTEN and INPP5D bacterial growth assays: The authors interpret the difference in growth as measured by luminescent reporter vs. CFU as a difference in assay sensitivity. However, the CFU assay at 4 days is at risk of being skewed by differential cell death because of the wash step prior to plating that would remove bacteria released to the media when cells die. Given that the INPP5D knockout seems to enhance inflammatory pathways, which can in turn contribute to cell death, this would be an alternate explanation for the discrepancy in growth phenotypes. The authors have previously used cell survival/death assays in parallel with CFU (as in reference 25), and that would be helpful in distinguishing whether the modest decrease in bacterial burden observed is truly due to decreased intracellular bacterial growth or enhanced cell death.

Routinely, we no longer perform cell viability assays before plating CFUs on day 4. This is because we have already extensively optimized our infection assays in BMDM^h^s to the extent where we consistently achieve >95% cell survival on day 4, with minimal cell death regardless of the genotypes of the macrophage mutants involved. To achieve this, we infect macrophage monolayers at very low MOIs (typically 0.3-0.4 for CFU assays and 0.5 for luminescence assays) to avoid both overactivation and Mtb-induced cytotoxicity. We have added additional text detailing our experimentally validated rationale (Lines 624-630) together with the references to our previous studies in which these specific experiments were performed (PMIDs: 39472457, 40080408, 40934365).

**Reviewer #2**: Lines 704-705. Please indicate the methods for data normalization and adjustment of p values, if performed, in order to account for variability across independent biological replicate experiments. Although a figure in Reference 84 includes adjusted p value, it is unclear whether adjusted or unadjusted p values are reported for the proteomics analysis in the present manuscript under review (S1 Data, Fig. 1, 2, 4). This is important to clarify given the authors conclusions about the magnitude of changes underscoring the central roles for GID/CTLH (line 329).

As requested by the reviewer we have now provided a detailed description of the data normalization strategy in the revised manuscript (lines 705-707). For hit selection, we used unadjusted p-values in combination with effect sizes (log2FC), as described in lines 704-705. This approach has been widely used in several published proteomic studies across immunology and other research fields (example PMIDs: 36450731, 32058041, 36384108, 41016070, 36215033, 40631140, 19605365). In these cases, FDR correction or adjusted p-values are often not applied to minimize the exclusion of relevant hits, as MS-based proteomics is subject to considerable technical variation arising during both sample preparation and mass spectrometry acquisition, and the application of stringent FDR thresholds can result in the removal of otherwise biologically significant hits (PMIDs: 27461997, 28802010). We were, however, able to apply FDR correction during database search by supplementing the search databases with decoy and contaminant sequences, as is standard practice (lines 685-687). We additionally selected a relatively low log2FC threshold because our primary objective was to perform downstream analyses of enriched biological pathways at the global proteome level. This strategy is consistent with our previous transcriptomic analyses (PMIDs: 40080408, 39472457) and is also common practice in numerous proteomic studies (example PMID: 32058041).

The authors used a log2 fold change of 0.3 (or absolute fold change of 1.23) and p value <0.05 as statistically significant, which is more generous than what others have published. A log2 fold change of 0.3 is only a 1.23-fold absolute difference, which is modest. In Reference 34, the criteria for statistical significance were more stringent (log2-fold change >2 and p-value <0.01). Furthermore, additional previously published proteomic analyses (referenced below under Minor criticism) used a log 2-fold change cutoff of 1 and adjusted p value <0.05.

See our response in the comment above.

The authors present conflicting data in the Figure 4B luminescence versus CFU results. S5 data and Fig 6A show that 2 edited cell lines for each PTEN and INPPD5 were generated. However, the growth assays in Fig 6 and the blots in Fig 7 only showed results from the guide 1 cells. The conclusion that PTEN- or INPPD5-deficiency restricts Mtb growth would be strengthened if the results from the edited cells using guides 1 and 2 both showed consistent phenotypes. To clarify the reproducibility of these results, please indicate in the Fig. 6 legend how many independent experiments were performed. These additional experiments would help clarify whether PTEN- or INPP5D-deficiency consistently reduces Mtb growth across different guides and in more than one experiment.

We acknowledge the reviewer’s concern and, as noted in lines 438-443 of the manuscript, discrepancies between luminescence and CFU-based growth measurements are commonly observed due to differences in the relative sensitivity of the two different assays. For each target gene, we typically generate two mutants carrying independent sgRNAs to account for potential differences in sgRNA potency. Both sgRNAs are included in the initial primary phenotypic assessment, whereas a representative sgRNA is used for subsequent analyses. When the knockout efficiencies are comparable, the resulting phenotypes are generally indistinguishable. Therefore, to avoid data redundancy and maintain consistency with our previous studies (PMIDs: 39472457, 40080408, 40934365), we usually report data from a representative sgRNA, not all sgRNAs. We have added a sentence and references to clarify this (Lines 1020-1022). As shown in the original Fig 6B below (CFU data), growth kinetics were largely similar between mutants generated with either sgRNA. The numbers of biological replicates for both CFU and luminescence measurements are provided in the Fig 6 legend.

**Reviewer #3**: MAJOR CONCERNS
1) Direct vs indirect effects of GID complex KO are unclear in several analyses. In their prior work this group showed widespread transcriptional effects in GID8 or MAEA KO cells with >1200 genes altered. It is unclear from their current data how many of the changes in protein expression they observe are due to transcriptional effects rather than the assumed change in protein stability. Indeed, they observe as many increased di-Gly peptides as they do decreased di-Gly peptides – arguing that indirect effects are having a major impact on the di-Gly changes. In addition, it is hard to know how many of the di-Gly changes seen here are due to changes in actual ubiquitylation vs simply being changes in overall protein level. For example, in their data PTEN has a modest -0.46 log2 change in di-Gly abundance but also has a -0.36 change in total protein - thus there is virtually no change in Ub state of the peptide. While the authors provide a rationale for why they chose not analyze protein vs PTM changes, this approach lacks rigor and precludes identifying those sites that are actually undergoing differential modification. This is the reason that it is standard practice in most PTM proteomic papers to perform an additional analysis that integrates protein level changes and peptide PTM change (for example PMID: 21906983, PMID: 35417684).

We agree with the reviewer that it is difficult to tease out direct versus indirect effects of the GID/CTLH complex, even with the proteomic analyses presented in our latest manuscript, given the broad involvement of the complex and its subunits in several different biological pathways. Moreover, linking transcriptional changes to protein expression is usually not always correlative due to differences in the rates of mRNA degradation and/or protein synthesis. We decided to perform proteomic analyses for this reason as although we observed some candidate antibacterial effectors in infected GID/CTLH knockout macrophages identified through RNA-seq, we recognized that some effectors could only be revealed at the proteomic level, since the complex largely and primarily operates at a post-translational level.

As we mention in the manuscript, for any individual protein, total protein abundance does not correlate well with changes in di-Gly peptides. Several other studies have, indeed, attempted to perform additional analyses integrating protein-level changes and peptide PTMs, however it is becoming increasingly apparent that these correlations are frequently problematic, particularly in ubiquitylation studies, as has been discussed recently by Mayor and colleagues (PMID: 41579396). Using PTEN data as an example, although it appears that there is a modest change in di-Gly signal that could quickly be assumed to be a consequence of reduced protein abundance, our CHX chase assay revealed that it is in fact stabilized when the GID/CTLH complex is knocked out (Fig 5C), confirming it as a candidate ubiquitylated substrate. So, while we agree with the reviewer that such a correlation could be of value, it has proven to be challenging and problematic, specifically in ubiquitylation analyses.

2) In their prior work they carried out metabolic analysis of GID8 and MAEA KO cells. Here they suggest that PTEN and SHIP1 are important mediators of the GID/CTLH complex. However, there is little mechanistic support for this. They do not perform metabolic analysis as was done in their prior work to determine if the same metabolic processes are affected in PTEN or SHIP1 macrophages as the GID-deficient macrophages. They do examine growth of Mtb in PTEN and SHIP1 KO cells but there is virtually no phenotype in PTEN cells. Showing similar metabolic changes in their PTEN/SHIP1 mutant cells would help support the hypothesis.

We would like to clarify that we do not make any claims in the manuscript that PTEN and SHIP1 mediate GID/CTLH complex activity. While we agree with the reviewer that the phenotype of the PTEN and SHIP1 knockout cells is markedly weaker than that of macrophages with an inactive GID/CTLH complex, a point we mention in the manuscript (Lines 455-457), we instead emphasize that PTEN and SHIP1 are candidate substrates of the complex. Given the involvement of both proteins in inflammatory processes, it is unlikely that they can be directly linked to the antibacterial properties of macrophages lacking the complex, particularly since loss of the complex results in a strongly anti-inflammatory phenotype (Lines 426-431). Instead, our objective was to validate selected candidate substrates of the complex, specifically PTEN and SHIP1, by demonstrating that they are stabilized in GID/CTLH knockout macrophages in a proteasome-dependent manner. These proteins are also relevant to macrophage control of Mtb, as they emerged as primary hits in our previous CRISPR-Cas9 knockout screen (PMID: 39472457), a point we highlight in the manuscript (Lines 395-398). We appreciate the suggestion to examine the metabolic profiles of PTEN- and SHIP1-deficient cells and agree this could be interesting. However, for the reasons outlined above, we do not believe this analysis would meaningfully advance the central narrative of our story as these are included as “proof of principle” substrates.

3) Their experiments with the 3AC inhibitor seem to be greatly impacted by non-specific effects of 3AC. For example, their supplementary data shows that 3AC dramatically impaired Mtb growth directly in vitro, and the very small additional growth change seen in SHIP1 KO vs control macrophages (Fig 6C) is not likely not to be biologically significant. The concerns about specificity are underscored by their transcriptomic analysis that shows the mutant cells and 3AC-treated cells clustering far apart. Overall I do not think that the 3AC inhibitor studies provide much supporting evidence for their underlying hypothesis.

We agree completely with reviewer's concern regarding the pleiotropic effects of 3AC on Mtb growth in broth culture. We refer the reviewer to our response to the same concern raised in the first major comment from Reviewer 1, where we address this issue in detail. We have reworded the corresponding result section extensively in the revised manuscript to acknowledge the caveats attached to these data.

**Part III – Minor Issues: Editorial and Data Presentation Modifications**
Please use this section for editorial suggestions as well as relatively minor modifications of existing data that would enhance clarity.

**Reviewer #1:** -INPP5D was presumably selected as a key substrate of CTLH complex ubiquitin ligase activity because it was thought to potentially be an intermediate effector of CTLH activity, but loss of INPP5D seems to have the opposite effect of the predicted effect. This should ideally be discussed a bit more.

Our selection of INPP5D and PTEN for validation was not based on a hypothesis that they function as intermediate effectors of GID/CTLH complex activity. Rather, INPP5D was selected because it was among the top hits identified in our ubiquitylome analysis, as it exhibited several downregulated diGly peptide sites (Fig 4C). In addition, INPP5D, together with PTPN6 and PTEN, belong to a group of inhibitory phosphatases that emerged as potential host determinants of Mtb growth in macrophages in our previous CRISPR-Cas9 screening study (PMID: 39472457). We inserted clearer justification for their selection in the manuscript (lines 395-398).

-Figure 2A- given the significant overlap in the differentially abundant proteomes between mutants, the shape of the Venn Diagram could be modified to represent this overlap

We have modified the Venn diagram to highlight the overlapping features in bold and larger text.

-Figures 1 and 2 could be combined and 2B could be supplementary as it primarily shows the distribution of individual replicates

We believe this would overly complicate Fig 1. We had combined Fig 2A with Fig 2B to highlight that the commonly abundant proteins in the Venn diagram can also be visualized, albeit indirectly, via the heatmap in Fig 2B as we narrate it in the results section.

-line 270 may be missing the word “knockout”

We thank the reviewer for identifying this. The word “knockout” has been added.

-this work is carried out in mouse macrophages, and whether CTLH has similar effects in human macrophages is not tested. I think testing in human knockout macrophages may be beyond the scope of this work, but it would be reasonable to mention this point as a potential limitation in the discussion section.

We thank the reviewer for pointing this out. We discuss these limitations, albeit for all macrophage populations in vivo, in lines 573-582 of the manuscript.

**Reviewer #2**: Could the authors comment on whether the ubiquitylated residues in the two phosphatases that they chose to focus on in this manuscript (displayed in Fig 4C) have been previously reported in the literature as known post-translationally modified sites in these proteins, or if they are novel post-translational modifications?

To the best of our knowledge, we are not aware of any reports describing the exact lysine residues identified in these two phosphatases. However, for PTEN, several other lysine positions that are ubiquitylated by different E3 ligases have been reported and are reviewed elsewhere (PMID: 32984016). We have added mention of this to the manuscript (Lines 418-420).

Fig 3A, Fig 7C. I recommend the authors highlight, bold, or color code the names of the pathways that they call out in the text of their manuscript so the reader can more quickly assess how the fold enrichment and counts for these pathways compare in context to the other pathways displayed in the figures.

We thank the reviewer for this point. We have highlighted the pathways that are of emphasis in the revised figures and figure legends.

Line 322. Additional global host diGly-proteomic profiling experiments during Mtb infection of macrophages were performed (doi: <https://doi.org/10.1101/2020.05.27.116772> and PMID 31951200), but the authors did not cite them.

We thank the reviewer for identifying this oversight and apologize for not citing this work in the original manuscript. These references have now been included in the revised version.

Lines 456-457, 483, and others. Complementation experiments were not performed in the PTEN and INPPD mutants to demonstrate that effects were from PTEN and INPP5D per se. Rather, the data presented show that deficiency of PTEN or deficiency of INPP5D produces phenotypes such as reduced intracellular Mtb growth. Similarly, Line 329 would be more accurately rephrased to describe that the deficiency or lack of GID/CTLH produces changes in the macrophage proteome during infection.

We have removed the words "confirm" and "underscore" from Lines 456-457, 483, and 329 to avoid inferring direct causality, given the absence of complementation studies, as the reviewer rightly points out. These changes are highlighted in Lines 455, 482 and 330 of the revised manuscript.

Line 480. Enrichment instead of enriched.

This has been corrected.

Lines 714-715. Please include the dilutions of the antibodies.

These have been included

**Reviewer #3:** • Several instances where data are overinterpreted or mis-stated.
o Line 535 Our diGly ubiquin-remnant profiling revealed that the GID/CTLH complex directly targets a remarkably wide spectrum of substrates, Their data does not distinguish between direct and indirect effects, and as discussed above, their data actually suggest the opposite – that there are a large number of indirect effects.

We thank the reviewer for identifying and pointing this out. We have changed the word "direct" to "appears to" to reflect the fact that some of the identified hits may indeed be indirect targets (Line 534). We have also removed several instances of the term "direct" in the manuscript to avoid implying direct regulation and to better reflect this limitation.

o Line 146 inhibitory phosphatases PTEN and INPP5D as proteasome-degraded substrates. They show no data demonstrating that these are direct substrates such as direct binding data in cells or in vitro ubiquitylation assays.

We thank the reviewer for this important comment. We agree that our current data do not demonstrate that PTEN and INPP5D are direct substrates of the GID/CTLH complex, as we have not performed direct binding experiments or *in vitro* ubiquitylation assays. Our conclusions are based on cellular evidence indicating that PTEN and INPP5D protein levels are regulated in a GID/CTLH-dependent manner and that their degradation occurs through the proteasome. To avoid overstating our findings, we have revised the text to describe PTEN and INPP5D as “putative” proteasome-degraded targets of the GID/CTLH complex (Line 146).

o Line 470 Pharmacologic inhibition of INPP5D using 3AC similarly enhanced Akt phosphorylation. In Figure 7A the 3AC treated cells actually have decreased p-Akt

In Fig 7A, 3AC does not decrease Akt phosphorylation. The level of phosphorylation in untreated, Mtb-infected macrophages appears to be higher than that in Mtb-infected macrophages treated with 3AC. We believe this is primarily due to the direct anti-Mtb effect of 3AC, which reduces the extent of macrophage activation by viable bacteria. However, in both conditions, the level of Akt phosphorylation remains higher than that observed in untreated, uninfected macrophages. This interpretation is supported by the replicate blot shown in S7A Fig.

• Very unusual biochemical conditions would benefit from clarification. They describe the binding conditions for their antibody as “Peptides were dissolved in enrichment buﬀer (80% acetonitrile/6% trifluoroactic acid saturated with glycine) and incubated with pre-washed antibody-conjugated beads on a rotating platform.” It is unclear how any protein would maintain activity in a solution of 80% organic solvent at pH <2. Can the authors provide references and/or rationale for using this as the binding condition for an immunoprecipitation experiment?

We thank the reviewer for raising this point. We agree that these conditions are unusual when compared with conventional protein immunoprecipitation protocols. However, this enrichment is performed at the peptide level rather than the protein level and follows both the manufacturer's recommended protocol and an internally validated protocol for the immunoaffinity enrichment of K-ε-GG-modified peptides (PTM Biolabs and Creative Proteomics). The high organic solvent concentration and acidic conditions are intended to minimize non-specific peptide binding while preserving the interaction between the antibody and the diGly epitope on peptides, thereby improving enrichment specificity. The Creative Proteomics core facility that processed the samples is referenced in the manuscript (Lines 643-644), and the enrichment procedure was performed according to their established workflow. We now draw the reviewer's attention to these details to clarify that these atypical conditions are recommended for the enrichment of these ubiquitinated peptides.

• They do not actually show the protein and di-Gly changes for SHP1 and PTEN in any of the figures.

We have included data that show protein-level changes for both INPP5D and PTEN by Western blot, together with quantitative analyses, in Fig 5A, 5C, and S5A-5B, 5D Figs. We do not have access to modification-specific antibodies to quantify the individual diGly modifications; therefore, we used cycloheximide (CHX) chase assays as a surrogate measure of total protein abundance and stability in the presence and absence of the GID/CTLH complex.

• Hard to read the data in several figures, for example the Venn Diagrams and pathway analysis.

We have increased the font size in the Venn diagrams, highlighted the pathways of interest in the pathway analysis figures, and improved the resolution of all figures throughout the manuscript.
