# Supplementary material for: Ubiquitylation by the GID/CTLH complex regulates the metabolic and innate immune response of macrophages to infection by Mycobacterium tuberculosis
Source: PLoS Pathog. 2026 Sep 18;22(9):e1014196. doi: 10.1371/journal.ppat.1014196 (PMC13614649; doi:10.1371/journal.ppat.1014196)
Supplement: Supplementary file 3 [file ppat.1014196.s021.docx]

**Reviewers' Comments:**

Reviewer's Responses to Questions

**Part I - Summary**
Please use this section to discuss strengths/weaknesses of study, novelty/significance, general execution and scholarship.

Reviewer #1: In this revised manuscript, Simwela et al have made minor changes to the initial manuscript but have not fundamentally addressed the few significant points raised by the three reviewers. The argument that there's justification to include the 3AC data is reasonable, and additional information is included about statistical analysis. Other points raised by reviewers (lack of experimental validation of proteomics data (R1), lack of cell viability assays (R1), lack of inclusion of data for both guide sgRNAs (R2), non-standard/suboptimal analytical approach (R3) are not experimentally addressed, and the written justification offered is not sufficiently compelling to circumvent the need for experimentally addressing those points.

We understand the reviewer’s concerns and thank them for taking the time to re-review our revised manuscript. We have now added new data and reanalyzed existing data to address these concerns in the sections below or, where appropriate, in our responses to the specific comments raised by Reviewers 2 and 3.

Reviewer #2: I appreciate that the authors shared the original version of Figure 6 showing the data from the second PTEN and INPP5D guides. The authors addressed my previous concerns raised about the statistical analysis of the proteomics data and the rationale for performing Mtb growth assays in only one of the knockout lines.

We thank the reviewer for the acknowledgement. Nevertheless, we have decided to include data for both sgRNAs in the revised Fig 6B, as this point has also been raised by Reviewer 1. We have clarified in the figure legend that, given the similarity in phenotypes between the two mutants, a representative sgRNA was used for the subsequent experiments.

Reviewer #3: The study by Simwela et al seeks to understand how ubiquitiylation regulates macrophage-intrinsic responses to Mycobacterium tuberculosis (Mtb). It follows up on the findings of an elegant genome-wide CRISPR screen that identified components of the GID/CTLH E3 ligase complex as important in regulating metabolism and restricting Mtb growth in macrophages. They carry out several quantitative MS experiments to profile changes in these cells and then follow-up with biochemical analyses of several selected proteins and inhibitors.

There are several strong aspects to the current study including in-depth proteomic analysis of both total protein and di-Gly modifications in GID-deficient cells with careful bioinformatic pathway enrichment analysis to identify processes that may be regulated by GID. The total protein analysis is rigorous and they were able to profile a large fraction of the expressed proteome with rigorous quantification. Notably, their pathway analysis demonstrates a convincing enrichment of core metabolic processes involved in ATP generation in KGID/CTL KO macrophages. Their CHX-chase experiments are also convincing, and demonstrate stabilization of SHIP1 and PTEN.

However there are several aspects of this study that are not as well developed. In some cases the proteomic analyses are missing important analyses that introduce confounding variables and the PTM data hard to interpret. In addition, most of the data are descriptive analyses of proteome or transcriptome changes in mutant cells or inhibitor-treated cells, with few mechanistic experiments included. When there are specific hypotheses tested many of the phenotypes are small, such as the changes in Mtb growth in mutant macrophages. Finally, there are methodologic concerns about some of the experiments. The above concerns are detailed below:

We provide our responses to the reviewers’ comments and concerns in the corresponding sections below. In brief, we have included additional proteomic analysis and clarification of our methodologies.

**Part II – Major Issues: Key Experiments Required for Acceptance**
Please use this section to detail the key new experiments or modifications of existing experiments that should be absolutely required to validate study conclusions.

Generally, there should be no more than 3 such required experiments or major modifications for a "Major Revision" recommendation. If more than 3 experiments are necessary to validate the study conclusions, then you are encouraged to recommend "Reject".

Reviewer #1: The same major issues persist from the first submission.

Reviewer #2: The authors adequately addressed my previous concerns. I do not recommend additional experiments.

Reviewer #3: The authors did no additional experiments for the revised submission and I find their responses to most issues previously raised by the reviewers did not adequately address the issues, as detailed below.

PRIOR CONCERNS FROM OTHER REVIEWERS
R1
1) Non-specificity of 3AC – not addressed. They did not address the important concern of R1 on the lack of specificity of 3AC effect. 3AC seems to have major non-specific effects in this system at the concentrations they used, including on Mtb itself. Their data in Fig 6C contradicts their model and contradicts the arguments for specificity they make to in response to R1 concerns. In INPP5D KO cells Mtb grows slightly worse (Fig 6B). However in 3AC treated KO cells bacteria grow slightly better than 3AC- treated control cells. Rather than phenocopying the KO, it has the opposite effect. I agree with the concerns of R1 in this regard.

Although we initially were of the opinion that the 3AC data had value given the widespread use of 3AC as an INPP5D inhibitor, we understand the reviewer’s concern regarding its “off-target” effects on the bacteria. In view of these comments, we have removed all 3AC-related data from the latest revised manuscript. This includes Fig 6C, the 3AC immunoblot panels in Fig 7A, and the transcriptomic data from infected, 3AC-treated macrophages previously presented in Fig 7B and S8-S9 Figs. We have retained the broth activity data for 3AC against Mtb (S8 Fig, previously S6 Fig), which is referred to briefly in the revised manuscript. We now state that we attempted to use 3AC to validate the role of host macrophage INPP5D, but the compound’s anti-Mtb activity limited its usefulness (Lines 483-486).

2) Differential cell death of mutant macrophages – not addressed. R1 was also concerned in differential cell death effecting Mtb growth phenotypes in ex vivo macrophages. I agree with this. Differential cell death in genetically manipulated macrophages can be a major confounder, particularly in this setting where PI3K and AKT are known regulators of apoptosis and cell death. R1 suggested a fairly straightforward analysis of cell death in the KO cells relative to control cells. However, the authors chose not to do this and argued that they do not need to because they have not seen increased cell death in prior studies with other knockouts. This is completely unconvincing as different genetic knockouts will have different specific phenotypes with some altering cell death and others not.

We apologise for not spelling out what we had done as cell death controls to measure cytotoxicity and viability in Mtb-infected mutant macrophages, and for not including a coherent set of data addressing this concern in the previous version of the manuscript. We agree fully that cell death is an important potential confounder when interpreting intracellular bacterial burden. For each mutant line generated, we implement multiple controls to assess cell viability and cytotoxicity in macrophage mutants infected with Mtb at different MOIs and for different durations. These include microscopic examination, LDH release assays, and live/dead cell viability assessment by flow cytometry. By the time we perform CFU assays or other experiments, we have a detailed understanding of the cell death kinetics of the mutants under investigation following infection with Mtb at different MOIs and for specific infection durations. As part of these internal controls, we routinely monitor macrophage monolayers microscopically to ascertain that there is no noticeable evidence of significant cell death prior to plating CFUs or performing any other experimental readout. Accordingly, we have an extensive dataset assessing Mtb-induced cytotoxicity and cell viability for the macrophage mutants used in this study.

We had regarded these as routine internal controls and therefore cited our previous studies rather than presenting the data for each mutant directly in this manuscript. This was an error on our part, and we appreciate that the inclusion of a full, integrated dataset showing cell viability data at time points corresponding to CFU enumeration is an important addition to the manuscript. Therefore, in response to the reviewer’s comment, we have now included relevant cell survival (live/dead) data measured by flow cytometry for INPP5D^−/−^, GID8^−/−^, and MAEA^−/−^ macrophages infected with Mtb at a comparable MOI and for the same duration as used in the CFU experiments (new S7 Fig). These data support our contention that the differences in bacterial burden observed between the mutant and control macrophages cannot be readily attributed to differential host-cell survival. We have also incorporated a description of these new data in the revised manuscript (Lines 476-483, 766-771).

MY PRIOR CONCERNS
1) Mild phenotype/little mechanistic support – not addressed. The critical validation of the functional importance of PTEN/INPP5D in host defense against MTB relies on analysis of ex vivo BMDMs at a single timepoint (Fig 6B). This shows a 20% reduction in CFU in PTEN KO cells and 2-fold reduction in INPP5D KO cells. These are very mild phenotypes and do not argue strongly for the importance of these proteins in MTB control, especially in comparison to the author’s very compelling prior work on the GID/CTLH where 1) the Mtb growth phenotpe was stronger and 2) was supported by strong additional mechanistic data showing changes in metabolism. Unlike their prior work, which had extensive metabolic profiling, this study has very little mechanistic analysis of what PTEN/INPP5D are doing in this context, aside from their long-known role in modulating PI3K/AKT pathway activity. They do perform RNA-Seq on KO cells and identify numerous changes in inflammatory mediators but it is unclear which of them are important in this context. The role of PI3K/AKT in suppressing inflammation during Mtb infection has also already been well-described (PMID: 26166760; PMID: 30356420; PMID: 33469941). Thus, in its current form, this work rests on a mild phenotype at a single timepoint with little mechanistic insight gained - making it an incremental advance over prior studies at this time.

We agree that the PTEN and INPP5D phenotypes are more modest than that observed following loss of the GID/CTLH complex, and we clarify this distinction in the manuscript (Lines 576-592). Importantly, PTEN and INPP5D were selected in part because they were independently identified as hits with potential roles in host control of Mtb in our previous CRISPR-Cas9 screen (PMID: 39472457; Lines 410-413) and in our diGly proteomics datasets as potential GID/CTLH ubiquitylation substrates, providing complementary evidence for their prioritization for functional characterization. We subsequently showed that both proteins are stabilized in GID/CTLH-deficient macrophages in a proteasome-dependent manner, and that their individual deletion alters Mtb growth, albeit to modest degrees. Thus, these experiments were not intended to establish PTEN or INPP5D as major standalone regulators of Mtb growth or to phenocopy loss of the entire GID/CTLH complex, but rather to provide functional validation of candidate substrates of the GID/CTLH complex that could have important independent roles in host control of Mtb. Given that we fully acknowledge that the phenotype in the PTEN/INPP5D mutants is modest and, while consistent, does not account for the strong phenotype observed in the GID/CTLH mutants, we see limited value in pursuing the mechanism further in the PTEN/INPP5D arc. We envision the GID/CTLH phenotype to be complex, and we stress the point of not overselling the mechanistic involvement of PTEN/INPP5D in the Discussion section of the manuscript (lines 576-592).

2) Proteomics analysis not rigorous – not addressed. In the original submission I had raised the concern that they were interpreting changes in di-Gly abundance without controlling for the protein-level changes. They did not address this concern at all. Frankly this is a standard analysis in the proteomics field and it is unclear why the authors do not wish to carry out this analysis. In their response they cite a meta-analysis arguing that di-Gly and protein levels are not globally correlated. We agree with that assertion but it is irrelevant to whether a PTM change on any particular protein is due to a change in PTM stoichiometry or it is simply the expression level of the protein changing.

We have performed and included additional analyses examining the correlation between protein and diGly peptide abundance, as well as the statistical relationship between changes in protein and diGly peptide abundance, as requested by the reviewer. These new data are now included in S2 Fig. Although we observed a moderate correlation between protein and diGly peptide abundance changes, our overall analyses indicate that the majority of diGly changes are not explained by changes in protein abundance. This observation is consistent with findings from several previous studies, including the selected examples cited here (PMID: 41843626, 21906983, 41579396, 31138642, 29967540, 34321666). We have cited these studies reporting similar observations and rewritten the corresponding section of the Results to incorporate our additional analyses and provide better context (Lines 331-352). We had not included this additional analysis in the previous versions of the paper because of the arguments presented in the references above but accept fully that there is value in including both analytical pipelines in the manuscript, and have added it to the current version.

**Part III – Minor Issues: Editorial and Data Presentation Modifications**
Please use this section for editorial suggestions as well as relatively minor modifications of existing data that would enhance clarity.

Reviewer #1: (No Response)

Reviewer #2: None

Reviewer #3: Prior Concerns:
2) PTEN/iNPPD5 di-Gly and protein level changes not shown in figures. Not addressed. Since much of the study centers on these proteins, these data should either be clealy highlighted in their volcano plots or otherwise displayed in the main figures so that readers can readily see the degree to which the di-Gly and protein levels are changing. It should also be noted in the results section of the text.

We have highlighted the PTEN/diGly changes, including the specific modified lysine residues, in the revised Fig 4, particularly Fig 4B. These changes are also highlighted in Fig 4C. We have added further descriptions of these findings, including the corresponding changes at the protein level, to the Results section (Lines 413-420, 423-427, 436-442).

1) Very unusual immunoprecipitation conditions in organic solvent at pH~1.5 – addressed. While these are very odd conditions it seems the authors sent the samples for IP and MS analysis to Creative Proteomics. They added a sentence regarding this in the macrophage infection paragraph of the methods methods but this should be included in the MS-related sections of the methods as well that the analysis was actually done by Creative Proteomics and not the authors.

Text has been added to the MS methods section to clarify that MS processing, and database searching was carried out by Creative Proteomics (Lines 716-717).
