# Supplementary material for: Ubiquitylation by the GID/CTLH complex regulates the metabolic and innate immune response of macrophages to infection by Mycobacterium tuberculosis
Source: PLoS Pathog. 2026 Sep 18;22(9):e1014196. doi: 10.1371/journal.ppat.1014196 (PMC13614649; doi:10.1371/journal.ppat.1014196)
Supplement: Peer review history

# Peer review: Decision Letter 0

**DOI:** 10.1371/journal.ppat.1014196.r001

**Authors:** Helena Boshoff

21 May 2026

Ubiquitylation by the GID/CTLH complex regulates the metabolic and innate immune response of macrophages to infection by Mycobacterium tuberculosis

PLOS Pathogens

Dear Dr. Russell,

Thank you for submitting your manuscript to PLOS Pathogens. After careful consideration, we feel that it has merit but does not fully meet PLOS Pathogens's publication criteria as it currently stands. Therefore, we invite you to submit a revised version of the manuscript that addresses the points raised during the review process.

Please submit your revised manuscript by Jul 20 2026 11:59PM. If you will need more time than this to complete your revisions, please reply to this message or contact the journal office at plospathogens@plos.org. When you're ready to submit your revision, log on to https://www.editorialmanager.com/ppathogens/ and select the 'Submissions Needing Revision' folder to locate your manuscript file.

Please include the following items when submitting your revised manuscript:

* A letter that responds to each point raised by the editor and reviewer(s). You should upload this letter as a separate file labeled 'Response to Reviewers'. This file does not need to include responses to any formatting updates and technical items listed in the 'Journal Requirements' section below.

* A marked-up copy of your manuscript that highlights changes made to the original version. You should upload this as a separate file labeled 'Revised Manuscript with Track Changes'.

* An unmarked version of your revised paper without tracked changes. You should upload this as a separate file labeled 'Manuscript'.

We look forward to receiving your revised manuscript.

Kind regards,

Helena Ingrid Boshoff

Section Editor

PLOS Pathogens

Helena Boshoff

Section Editor

Editor-in-Chief

PLOS Pathogens

orcid.org/0000-0003-2946-9497

Editor-in-Chief

PLOS Pathogens

orcid.org/0000-0002-7699-2064

**Journal Requirements:**

If the reviewer comments include a recommendation to cite specific previously published works, please review and evaluate these publications to determine whether they are relevant and should be cited. There is no requirement to cite these works unless the editor has indicated otherwise.

1) Please ensure that the CRediT author contributions listed for every co-author are completed accurately and in full.

At this stage, the following Authors/Authors require contributions: David G. Russell. Please ensure that the full contributions of each author are acknowledged in the "Add/Edit/Remove Authors" section of our submission form.

The list of CRediT author contributions may be found here: https://journals.plos.org/plospathogens/s/authorship#loc-author-contributions

2) We ask that a manuscript source file is provided at Revision. Please upload your manuscript file as a .doc, .docx, .rtf or .tex. If you are providing a .tex file, please upload it under the item type u2018LaTeX Source Fileu2019 and leave your .pdf version as the item type u2018Manuscriptu2019.

3) We do not publish any copyright or trademark symbols that usually accompany proprietary names, eg ©,  ®, or TM  (e.g. next to drug or reagent names). Therefore please remove all instances of trademark/copyright symbols throughout the text, including:

- TM on pages: 18, and 19.

4) Please upload all main figures as separate Figure files in .tif or .eps format. For more information about how to convert and format your figure files please see our guidelines:

https://journals.plos.org/plospathogens/s/figures

5) Please upload a copy of  FIGURES 1-7 which you refer to in your text on pages [5, 6, 7, 9, 11, 12, and 13]. Or, if the figure is no longer to be included as part of the submission please remove all reference to it within the text.

6) Please amend your detailed Financial Disclosure statement. This is published with the article. It must therefore be completed in full sentences and contain the exact wording you wish to be published.

1) State what role the funders took in the study. If the funders had no role in your study, please state: "The funders had no role in study design, data collection and analysis, decision to publish, or preparation of the manuscript."

2) If any authors received a salary from any of your funders, please state which authors and which funders..

7) <carina-action-element class="ng-star-inserted">We have noticed that you have uploaded Supporting Information files [S1-S7 Data,  Figs S1-S9], but you have not included a complete list of legends. Please add a full list of legends for your Supporting Information files after the references list.</carina-action-element>

NOTE - You have uploaded supporting information legends as separate file, Kindly include in the manuscript after reference list.

**Reviewers' Comments:**

Reviewer's Responses to Questions

**Part I - Summary**

Please use this section to discuss strengths/weaknesses of study, novelty/significance, general execution and scholarship.

Reviewer #1: In this manuscript, the authors explore how the CTLH complex contributes to reshaping the proteome during Mtb infection in macrophages. This work builds directly upon previous work in which the authors identified CTLH in a screen to find host factors that impair control of Mtb intracellular infection. Using comparative proteomics, they identify significant metabolic rewiring of metabolism in cells lacking CTLH components GID8 or MAEA, including increased abundance of proteins in oxidative phosphorylation pathways, increased abundance of lipid and fatty acid catabolic pathway proteins, and increase in abundance of proteins associated with reactive oxygen species. Proteins associated with innate immune functions are decreased in abundance, suggesting dampened inflammation. As CTLH has E3 ligase activity, the authors then perform comparative analysis of ubiquitinylated proteins to identify candidate substrates that could mediate the observed effects. Among candidate substrates, they focus on PTEN and INPP5D, finding that loss of INPP5D enhances pro-inflammatory gene expression- of note, this is in contrast to the immune dampening effect of the CTLH complex. The use of comparative proteomic approaches to understand the contributions of individual host factors to the macrophage response to Mtb infection is both unusual and valuable, and the use of two distinct CTLH subunits in the analysis is a nice validation of key findings. On balance, the work is a meaningful contribution to our understanding of how macrophages respond to Mtb, specifically the role of the CTLH complex in regulation of the immune and metabolic states of the cells.

Reviewer #2: Global protein abundance and diGly proteomic analyses were performed in scramble control, GID8, and MAEA deficient Hoxb8 BMDMs following Mtb infection. This led the investigators to test the hypothesis that the loss of GID/CTLH activity regulates metabolic and innate immune pathways. The authors focused on two candidate GID/CTLH substrates: the inhibitor phosphatases INPP5D and PTEN. Cycloheximide chase assays and Mtb growth assays in inhibitor phosphatase mutant cells revealed that the phosphatases were degraded by proteosomes, confirmed that the phosphatases are candidate GID/CTLH substrates, and revealed that Mtb growth was restricted in phosphatase-deficient cells by CFU but not luciferase assays. Mtb-infected GID/CTLH-deficient displayed downregulation of pro-inflammatory genes. The manuscript’s strengths are that it provides important insights into host ubiquitin ligases and their substrate phosphatases that control Mtb growth in macrophages and inflammatory responses. Furthermore, detailed follow up mechanistic studies were performed on several of the hits from the global ubiquitin profiling assays. The authors used both pharmacologic and genetic strategies to perturb host phosphatases. A weakness is that the experiments are performed in engineered HoxB8 BMDMs, although these have been shown to display transcriptional responses to Mtb that are similar to unmodified primary BMDMs. Additional weaknesses are described below.

Reviewer #3: The study by Simwela et al seeks to understand how ubiquitiylation regulates macrophage-intrinsic responses to Mycobacterium tuberculosis (Mtb). It follows up on the findings of an elegant genome-wide CRISPR screen that identified components of the GID/CTLH E3 ligase complex as important in regulating metabolism and restricting Mtb growth in macrophages. They carry out several quantitative MS experiments to profile changes in these cells and then follow-up with biochemical analyses of several selected proteins and inhibitors.

There are several strong aspects to the current study including in-depth proteomic analysis of both total protein and di-Gly modifications in GID-deficient cells with careful bioinformatic pathway enrichment analysis to identify processes that may be regulated by GID. The total protein analysis is rigorous and they were able to profile a large fraction of the expressed proteome with rigorous quantification. Notably, their pathway analysis demonstrates a convincing enrichment of core metabolic processes involved in ATP generation in KGID/CTL KO macrophages. Their CHX-chase experiments are also convincing, and demonstrate stabilization of SHIP1 and PTEN.

However there are several aspects of this study that are not as well developed. In some cases the proteomic analyses are missing important analyses that introduce confounding variables and the PTM data hard to interpret. In addition, most of the data are descriptive analyses of proteome or transcriptome changes in mutant cells or inhibitor-treated cells, with few mechanistic experiments included. When there are specific hypotheses tested many of the phenotypes are small, such as the changes in Mtb growth in mutant macrophages. Finally, there are methodologic concerns about some of the experiments. The above concerns are detailed below:

**********

**Part II – Major Issues: Key Experiments Required for Acceptance**

Please use this section to detail the key new experiments or modifications of existing experiments that should be absolutely required to validate study conclusions.

Generally, there should be no more than 3 such required experiments or major modifications for a "Major Revision" recommendation. If more than 3 experiments are necessary to validate the study conclusions, then you are encouraged to recommend "Reject".

Reviewer #1: Unclear rationale for use of 3AC- Given that 3AC has pleiotropic effects that are difficult to disentangle, inhibiting both INPP5D and Mtb growth independent of the macrophage, it’s difficult to know how to interpret 3AC data. On balance, the 3AC data may add more confusion than clarity to the key messages of the manuscript, and I would favor removing the 3AC data.

No validation of the comparative proteomic data: The authors note that GID8 and MAEA proteins are depleted in their respective knockout conditions, but there is no other validation of the comparative proteomics findings. Given the heavy weight placed on pathway analysis of these data in the manuscript, some additional validation of at least a few differentially abundant proteins by Western blotting would increase confidence in those interpretations.

No cell viability assay in the PTEN and INPP5D bacterial growth assays: The authors interpret the difference in growth as measured by luminescent reporter vs. CFU as a difference in assay sensitivity. However, the CFU assay at 4 days is at risk of being skewed by differential cell death because of the wash step prior to plating that would remove bacteria released to the media when cells die. Given that the INPP5D knockout seems to enhance inflammatory pathways, which can in turn contribute to cell death, this would be an alternate explanation for the discrepancy in growth phenotypes. The authors have previously used cell survival/death assays in parallel with CFU (as in reference 25), and that would be helpful in distinguishing whether the modest decrease in bacterial burden observed is truly due to decreased intracellular bacterial growth or enhanced cell death.

Reviewer #2: Lines 704-705. Please indicate the methods for data normalization and adjustment of p values, if performed, in order to account for variability across independent biological replicate experiments. Although a figure in Reference 84 includes adjusted p value, it is unclear whether adjusted or unadjusted p values are reported for the proteomics analysis in the present manuscript under review (S1 Data, Fig. 1, 2, 4). This is important to clarify given the authors conclusions about the magnitude of changes underscoring the central roles for GID/CTLH (line 329).

The authors used a log2 fold change of 0.3 (or absolute fold change of 1.23) and p value <0.05 as statistically significant, which is more generous than what others have published. A log2 fold change of 0.3 is only a 1.23-fold absolute difference, which is modest. In Reference 34, the criteria for statistical significance were more stringent (log2-fold change >2 and p-value <0.01). Furthermore, additional previously published proteomic analyses (referenced below under Minor criticism) used a log 2-fold change cutoff of 1 and adjusted p value <0.05.

The authors present conflicting data in the Figure 4B luminescence versus CFU results. S5 data and Fig 6A show that 2 edited cell lines for each PTEN and INPPD5 were generated. However, the growth assays in Fig 6 and the blots in Fig 7 only showed results from the guide 1 cells. The conclusion that PTEN- or INPPD5-deficiency restricts Mtb growth would be strengthened if the results from the edited cells using guides 1 and 2 both showed consistent phenotypes. To clarify the reproducibility of these results, please indicate in the Fig. 6 legend how many independent experiments were performed. These additional experiments would help clarify whether PTEN- or INPP5D-deficiency consistently reduces Mtb growth across different guides and in more than one experiment.

Reviewer #3: MAJOR CONCERNS

1) Direct vs indirect effects of GID complex KO are unclear in several analyses. In their prior work this group showed widespread transcriptional effects in GID8 or MAEA KO cells with >1200 genes altered. It is unclear from their current data how many of the changes in protein expression they observe are due to transcriptional effects rather than the assumed change in protein stability. Indeed, they observe as many increased di-Gly peptides as they do decreased di-Gly peptides – arguing that indirect effects are having a major impact on the di-Gly changes. In addition, it is hard to know how many of the di-Gly changes seen here are due to changes in actual ubiquitylation vs simply being changes in overall protein level. For example, in their data PTEN has a modest -0.46 log2 change in di-Gly abundance but also has a -0.36 change in total protein - thus there is virtually no change in Ub state of the peptide. While the authors provide a rationale for why they chose not analyze protein vs PTM changes, this approach lacks rigor and precludes identifying those sites that are actually undergoing differential modification. This is the reason that it is standard practice in most PTM proteomic papers to perform an additional analysis that integrates protein level changes and peptide PTM change (for example PMID: 21906983, PMID: 35417684).

2) In their prior work they carried out metabolic analysis of GID8 and MAEA KO cells. Here they suggest that PTEN and SHIP1 are important mediators of the GID/CTLH complex. However, there is little mechanistic support for this. They do not perform metabolic analysis as was done in their prior work to determine if the same metabolic processes are affected in PTEN or SHIP1 macrophages as the GID-deficient macrophages. They do examine growth of Mtb in PTEN and SHIP1 KO cells but there is virtually no phenotype in PTEN cells. Showing similar metabolic changes in their PTEN/SHIP1 mutant cells would help support the hypothesis.

3) Their experiments with the 3AC inhibitor seem to be greatly impacted by non-specific effects of 3AC. For example, their supplementary data shows that 3AC dramatically impaired Mtb growth directly in vitro, and the very small additional growth change seen in SHIP1 KO vs control macrophages (Fig 6C) is not likely not to be biologically significant. The concerns about specificity are underscored by their transcriptomic analysis that shows the mutant cells and 3AC-treated cells clustering far apart. Overall I do not think that the 3AC inhibitor studies provide much supporting evidence for their underlying hypothesis.

**********

**Part III – Minor Issues: Editorial and Data Presentation Modifications**

Please use this section for editorial suggestions as well as relatively minor modifications of existing data that would enhance clarity.

Reviewer #1: -INPP5D was presumably selected as a key substrate of CTLH complex ubiquitin ligase activity because it was thought to potentially be an intermediate effector of CTLH activity, but loss of INPP5D seems to have the opposite effect of the predicted effect. This should ideally be discussed a bit more.

-Figure 2A- given the significant overlap in the differentially abundant proteomes between mutants, the shape of the Venn Diagram could be modified to represent this overlap

-Figures 1 and 2 could be combined and 2B could be supplementary as it primarily shows the distribution of individual replicates

-line 270 may be missing the word “knockout”

-this work is carried out in mouse macrophages, and whether CTLH has similar effects in human macrophages is not tested. I think testing in human knockout macrophages may be beyond the scope of this work, but it would be reasonable to mention this point as a potential limitation in the discussion section.

Reviewer #2: Could the authors comment on whether the ubiquitylated residues in the two phosphatases that they chose to focus on in this manuscript (displayed in Fig 4C) have been previously reported in the literature as known post-translationally modified sites in these proteins, or if they are novel post-translational modifications?

Fig 3A, Fig 7C. I recommend the authors highlight, bold, or color code the names of the pathways that they call out in the text of their manuscript so the reader can more quickly assess how the fold enrichment and counts for these pathways compare in context to the other pathways displayed in the figures.

Line 322. Additional global host diGly-proteomic profiling experiments during Mtb infection of macrophages were performed (doi: https://doi.org/10.1101/2020.05.27.116772 and PMID 31951200), but the authors did not cite them.

Lines 456-457, 483, and others. Complementation experiments were not performed in the PTEN and INPPD mutants to demonstrate that effects were from PTEN and INPP5D per se. Rather, the data presented show that deficiency of PTEN or deficiency of INPP5D produces phenotypes such as reduced intracellular Mtb growth. Similarly, Line 329 would be more accurately rephrased to describe that the deficiency or lack of GID/CTLH produces changes in the macrophage proteome during infection.

Line 480. Enrichment instead of enriched.

Lines 714-715. Please include the dilutions of the antibodies.

Reviewer #3: • Several instances where data are overinterpreted or mis-stated.

o Line 535 Our diGly ubiquin-remnant profiling revealed that the GID/CTLH complex directly targets a remarkably wide spectrum of substrates, Their data does not distinguish between direct and indirect effects, and as discussed above, their data actually suggest the opposite – that there are a large number of indirect effects.

o Line 146 inhibitory phosphatases PTEN and INPP5D as proteasome-degraded substrates. They show no data demonstrating that these are direct substrates such as direct binding data in cells or in vitro ubiquitylation assays.

o Line 470 Pharmacologic inhibition of INPP5D using 3AC similarly enhanced Akt phosphorylaon. In Figure 7A the 3AC treated cells actually have decreased p-Akt

• Very unusual biochemical conditions would benefit from clarification. They describe the binding conditions for their antibody as “Peptides were dissolved in enrichment buﬀer (80% acetonitrile/6% trifluoroactic acid saturated with glycine) and incubated with pre-washed antibody-conjugated beads on a rotating platform.” It is unclear how any protein would maintain activity in a solution of 80% organic solvent at pH <2. Can the authors provide references and/or rationale for using this as the binding condition for an immunoprecipitation experiment?

• They do not actually show the protein and di-Gly changes for SHP1 and PTEN in any of the figures.

• Hard to read the data in several figures, for example the Venn Diagrams and pathway analysis.

**********

PLOS authors have the option to publish the peer review history of their article (what does this mean?). If published, this will include your full peer review and any attached files.

If you choose “no”, your identity will remain anonymous but your review may still be made public.

**Do you want your identity to be public for this peer review?**  For information about this choice, including consent withdrawal, please see our Privacy Policy.

Reviewer #1: No

Reviewer #2: No

Reviewer #3: No

[NOTE: If reviewer comments were submitted as an attachment file, they will be attached to this email and accessible via the submission site. Please log into your account, locate the manuscript record, and check for the action link "View Attachments". If this link does not appear, there are no attachment files.]

**Figure resubmission:**

**Reproducibility:**

?>

# Author comment: Author response to Decision Letter 1

**DOI:** 10.1371/journal.ppat.1014196.r002

21 Jul 2026

Attachment Submitted filename: Response to Reviewers Comments v3.docx

# Peer review: Decision Letter 1

**DOI:** 10.1371/journal.ppat.1014196.r003

**Authors:** Helena Boshoff

4 Aug 2026

Ubiquitylation by the GID/CTLH complex regulates the metabolic and innate immune response of macrophages to infection by Mycobacterium tuberculosis

PLOS Pathogens

Dear Dr. Russell,

Thank you for submitting your manuscript to PLOS Pathogens. After careful consideration, we feel that it has merit but does not fully meet PLOS Pathogens's publication criteria as it currently stands. Therefore, we invite you to submit a revised version of the manuscript that addresses the points raised during the review process.

Please submit your revised manuscript by Oct 03 2026 11:59PM. If you will need more time than this to complete your revisions, please reply to this message or contact the journal office at plospathogens@plos.org. When you're ready to submit your revision, log on to https://www.editorialmanager.com/ppathogens/ and select the 'Submissions Needing Revision' folder to locate your manuscript file.

Please include the following items when submitting your revised manuscript:

* A letter that responds to each point raised by the editor and reviewer(s). You should upload this letter as a separate file labeled 'Response to Reviewers'. This file does not need to include responses to any formatting updates and technical items listed in the 'Journal Requirements' section below.

* A marked-up copy of your manuscript that highlights changes made to the original version. You should upload this as a separate file labeled 'Revised Manuscript with Track Changes'.

* An unmarked version of your revised paper without tracked changes. You should upload this as a separate file labeled 'Manuscript'.

We look forward to receiving your revised manuscript.

Kind regards,

Helena Ingrid Boshoff

Section Editor

PLOS Pathogens

Helena Boshoff

Section Editor

Editor-in-Chief

PLOS Pathogens

orcid.org/0000-0003-2946-9497

Michael Malim

Editor-in-Chief

PLOS Pathogens

orcid.org/0000-0002-7699-2064

**Additional Editor Comments (if provided):**

**Journal Requirements:**

If the reviewer comments include a recommendation to cite specific previously published works, please review and evaluate these publications to determine whether they are relevant and should be cited. There is no requirement to cite these works unless the editor has indicated otherwise.

<carina-action-element class="ng-star-inserted" style="color: rgba(0, 0, 0, 0.87); font-family: sans-serif; font-size: 12px;">1) We do not publish any copyright or trademark symbols that usually accompany proprietary names, eg ©, ®, or TM (e.g. next to drug or reagent names). Therefore please remove all instances of trademark/copyright symbols throughout the text, including:</carina-action-element>

<carina-action-element class="ng-star-inserted" style="color: rgba(0, 0, 0, 0.87); font-family: sans-serif; font-size: 12px;"></carina-action-element><carina-action-element class="ng-star-inserted">- ® on page: 17.</carina-action-element>

2) PLOS now requires that authors provide the original uncropped and unadjusted images underlying all blot or gel results reported in a submission’s figures or Supporting Information files. This policy and the journal’s other requirements for blot/gel reporting and figure preparation are described in detail at https://journals.plos.org/plosone/s/figures#loc-blot-and-gel-reporting-requirements and https://journals.plos.org/plosone/s/figures#loc-preparing-figures-from-image-files.

When you submit your revised manuscript, please ensure that your figures adhere fully to these guidelines and provide the original underlying images for all blot or gel data reported in your submission. See the following link for instructions on providing the original image data: https://journals.plos.org/plosone/s/figures#loc-original-images-for-blots-and-gels.

In your cover letter, please note whether your blot/gel image data are in Supporting Information or posted at a public data repository, provide the repository URL if relevant, and provide specific details as to which raw blot/gel images, if any, are not available. Email us at customercare@plos.org if you have any questions.

**Reviewers' Comments:**

Reviewer's Responses to Questions

**Part I - Summary**

Please use this section to discuss strengths/weaknesses of study, novelty/significance, general execution and scholarship.

Reviewer #1: In this revised manuscript, Simwela et al have made minor changes to the initial manuscript but have not fundamentally addressed the few significant points raised by the three reviewers. The argument that there's justification to include the 3AC data is reasonable, and additional information is included about statistical analysis. Other points raised by reviewers (lack of experimental validation of proteomics data (R1), lack of cell viability assays (R1), lack of inclusion of data for both guide sgRNAs (R2), non-standard/suboptimal analytical approach (R3) are not experimentally addressed, and the written justification offered is not sufficiently compelling to circumvent the need for experimentally addressing those points.

Reviewer #2: I appreciate that the authors shared the original version of Figure 6 showing the data from the second PTEN and INPP5D guides. The authors addressed my previous concerns raised about the statistical analysis of the proteomics data and the rationale for performing Mtb growth assays in only one of the knockout lines.

Reviewer #3: The study by Simwela et al seeks to understand how ubiquitiylation regulates macrophage-intrinsic responses to Mycobacterium tuberculosis (Mtb). It follows up on the findings of an elegant genome-wide CRISPR screen that identified components of the GID/CTLH E3 ligase complex as important in regulating metabolism and restricting Mtb growth in macrophages. They carry out several quantitative MS experiments to profile changes in these cells and then follow-up with biochemical analyses of several selected proteins and inhibitors.

There are several strong aspects to the current study including in-depth proteomic analysis of both total protein and di-Gly modifications in GID-deficient cells with careful bioinformatic pathway enrichment analysis to identify processes that may be regulated by GID. The total protein analysis is rigorous and they were able to profile a large fraction of the expressed proteome with rigorous quantification. Notably, their pathway analysis demonstrates a convincing enrichment of core metabolic processes involved in ATP generation in KGID/CTL KO macrophages. Their CHX-chase experiments are also convincing, and demonstrate stabilization of SHIP1 and PTEN.

However there are several aspects of this study that are not as well developed. In some cases the proteomic analyses are missing important analyses that introduce confounding variables and the PTM data hard to interpret. In addition, most of the data are descriptive analyses of proteome or transcriptome changes in mutant cells or inhibitor-treated cells, with few mechanistic experiments included. When there are specific hypotheses tested many of the phenotypes are small, such as the changes in Mtb growth in mutant macrophages. Finally, there are methodologic concerns about some of the experiments. The above concerns are detailed below:

**********

**Part II – Major Issues: Key Experiments Required for Acceptance**

Please use this section to detail the key new experiments or modifications of existing experiments that should be absolutely required to validate study conclusions.

Generally, there should be no more than 3 such required experiments or major modifications for a "Major Revision" recommendation. If more than 3 experiments are necessary to validate the study conclusions, then you are encouraged to recommend "Reject".

Reviewer #1: The same major issues persist from the first submission.

Reviewer #2: The authors adequately addressed my previous concerns. I do not recommend additional experiments.

Reviewer #3: The authors did no additional experiments for the revised submission and I find their responses to most issues previously raised by the reviewers did not adequately address the issues, as detailed below.

PRIOR CONCERNS FROM OTHER REVIEWERS

R1

1) Non-specificity of 3AC – not addressed. They did not address the important concern of R1 on the lack of specificity of 3AC effect. 3AC seems to have major non-specific effects in this system at the concentrations they used, including on Mtb itself. Their data in Fig 6C contradicts their model and contradicts the arguments for specificity they make to in response to R1 concerns. In INPP5D KO cells Mtb grows slightly worse (Fig 6B). However in 3AC treated KO cells bacteria grow slightly better than 3AC- treated control cells. Rather than phenocopying the KO, it has the opposite effect. I agree with the concerns of R1 in this regard.

2) Differential cell death of mutant macrophages – not addressed. R1 was also concerned in differential cell death effecting Mtb growth phenotypes in ex vivo macrophages. I agree with this. Differential cell death in genetically manipulated macrophages can be a major confounder, particularly in this setting where PI3K and AKT are known regulators of apoptosis and cell death. R1 suggested a fairly straightforward analysis of cell death in the KO cells relative to control cells. However, the authors chose not to do this and argued that they do not need to because they have not seen increased cell death in prior studies with other knockouts. This is completely unconvincing as different genetic knockouts will have different specific phenotypes with some altering cell death and others not.

MY PRIOR CONCERNS

1) Mild phenotype/little mechanistic support – not addressed. The critical validation of the functional importance of PTEN/INPP5D in host defense against MTB relies on analysis of ex vivo BMDMs at a single timepoint (Fig 6B). This shows a 20% reduction in CFU in PTEN KO cells and 2-fold reduction in INPP5D KO cells. These are very mild phenotypes and do not argue strongly for the importance of these proteins in MTB control, especially in comparison to the author’s very compelling prior work on the GID/CTLH where 1) the Mtb growth phenotpe was stronger and 2) was supported by strong additional mechanistic data showing changes in metabolism. Unlike their prior work, which had extensive metabolic profiling, this study has very little mechanistic analysis of what PTEN/INPP5D are doing in this context, aside from their long-known role in modulating PI3K/AKT pathway activity. They do perform RNA-Seq on KO cells and identify numerous changes in inflammatory mediators but it is unclear which of them are important in this context. The role of PI3K/AKT in suppressing inflammation during Mtb infection has also already been well-described (PMID: 26166760; PMID: 30356420; PMID: 33469941). Thus, in its current form, this work rests on a mild phenotype at a single timepoint with little mechanistic insight gained - making it an incremental advance over prior studies at this time.

2) Proteomics analysis not rigorous – not addressed. In the original submission I had raised the concern that they were interpreting changes in di-Gly abundance without controlling for the protein-level changes. They did not address this concern at all. Frankly this is a standard analysis in the proteomics field and it is unclear why the authors do not wish to carry out this analysis. In their response they cite a meta-analysis arguing that di-Gly and protein levels are not globally correlated. We agree with that assertion but it is irrelevant to whether a PTM change on any particular protein is due to a change in PTM stoichiometry or it is simply the expression level of the protein changing.

**********

**Part III – Minor Issues: Editorial and Data Presentation Modifications**

Please use this section for editorial suggestions as well as relatively minor modifications of existing data that would enhance clarity.

Reviewer #1: (No Response)

Reviewer #2: None

Reviewer #3: Prior Concerns:

2) PTEN/iNPPD5 di-Gly and protein level changes not shown in figures. Not addressed. Since much of the study centers on these proteins, these data should either be clealy highlighted in their volcano plots or otherwise displayed in the main figures so that readers can readily see the degree to which the di-Gly and protein levels are changing. It should also be noted in the results section of the text.

1) Very unusual immunoprecipitation conditions in organic solvent at pH~1.5 – addressed. While these are very odd conditions it seems the authors sent the samples for IP and MS analysis to Creative Proteomics. They added a sentence regarding this in the macrophage infection paragraph of the methods methods but this should be included in the MS-related sections of the methods as well that the analysis was actually done by Creative Proteomics and not the authors.

**********

PLOS authors have the option to publish the peer review history of their article (what does this mean?). If published, this will include your full peer review and any attached files.

If you choose “no”, your identity will remain anonymous but your review may still be made public.

**Do you want your identity to be public for this peer review?**  For information about this choice, including consent withdrawal, please see our Privacy Policy.

Reviewer #1: No

Reviewer #2: No

Reviewer #3: No

[NOTE: If reviewer comments were submitted as an attachment file, they will be attached to this email and accessible via the submission site. Please log into your account, locate the manuscript record, and check for the action link "View Attachments". If this link does not appear, there are no attachment files.]

**Figure resubmission:**

**Reproducibility:**

?>

# Author comment: Author response to Decision Letter 2

**DOI:** 10.1371/journal.ppat.1014196.r004

14 Aug 2026

Attachment Submitted filename: Response to reviewers comments_2nd round v2.docx

# Peer review: Decision Letter 2

**DOI:** 10.1371/journal.ppat.1014196.r005

**Authors:** Helena Boshoff

9 Sep 2026

Dear Dr. Russell,

We are pleased to inform that your manuscript, "Ubiquitylation by the GID/CTLH complex regulates the metabolic and innate immune response of macrophages to infection by Mycobacterium tuberculosis", has been editorially accepted for publication at PLOS Pathogens.

Before your manuscript can be formally accepted and sent to production, you will need to complete our formatting changes, which you will receive by email within a week. Please note that your manuscript will not be scheduled for publication until you have made the required changes.

IMPORTANT NOTES

(1) Copyediting and Proofreading: The corresponding author will receive a typeset proof for review, to ensure errors have not been introduced during production. Please review the PDF proof of your manuscript carefully, as this is the last chance to correct any errors. Please note that major changes, or those which affect the scientific understanding of the work, will likely cause delays to the publication date of your manuscript.

(2) Appropriate Figure Files: Please remove all name and figure # text from your figure files. Please also take this time to check that your figures are of high resolution, which will improve the readbility of your figures and help expedite your manuscript's publication. Please note that figures must have been originally created at 300dpi or higher. Do not manually increase the resolution of your files. For instructions on how to properly obtain high quality images, please review our Figure Guidelines, with examples at: http://journals.plos.org/plospathogens/s/figures.

We encourage you to use PACE (http://pace.apexcovantage.com/), a figure-checking tool which helps authors ensure all figures meet PLOS requirements so that figures can publish in the highest quality. PACE is a digital diagnostic and conversion tool for figure files. It will provide information about any failed check(s) and, if able, will automatically convert the figure into an acceptable file that passes quality checks.

To use PACE, you must first register as a user. Then, login and navigate to the UPLOAD tab, where you will find detailed instructions on how to use the tool.

(3) Striking Image: Please upload a striking still image to accompany your article if one is available (you can include a new image or an existing one from within your manuscript). Should your paper be accepted, this image will be considered for our monthly issue image and may also appear on our website to feature your article. Please upload this as a separate file, selecting "striking image" as the file type upon upload. Please also include a separate "Other" file with a caption, including credits and any potential copyright information. Please do not include the caption in the main article file. If your image is from someone other than yourself, please ensure that the artist has read and agreed to the terms and conditions of the Creative Commons Attribution License at http://journals.plos.org/plospathogens/s/content-license. Please note that PLOS cannot publish copyrighted images.

(4) Press Release or Related Media: If you or your institution will be preparing press materials for this manuscript, please inform our press team in advance at plospathogens@plos.org as soon as possible. We ask that you contact us within one week to plan ahead of our fast Production schedule. If you need to know your paper's publication date for related media purposes, you must coordinate with our press team, and your manuscript will remain under a strict press embargo until the publication date and time.

(5) Update your Profile Information: Now that your manuscript has been provisionally accepted, please log into Editorial Manager and update your profile, if needed. Go to http://www.editorialmanager.com/ppathogens, log in, and click on the "Update My Information" link at the top of the page. Please update your user information to ensure an efficient production and billing process.

(6) LaTeX users only: Our staff will ask you to upload a TEX file in addition to the PDF before the paper can be sent to typesetting, so please carefully review our Latex Guidelines http://journals.plos.org/plospathogens/s/latex in the meantime.

Thank you again for supporting Open Access publishing; we are looking forward to publishing your work in PLOS Pathogens.

Best regards,

Helena Ingrid Boshoff

Section Editor

PLOS Pathogens

Helena Boshoff

Section Editor

PLOS Pathogens

Dominique Soldati-Favre

Editor-in-Chief

PLOS Pathogens

***********************************************************

Reviewer's Responses to Questions

**Part I - Summary**

Please use this section to discuss strengths/weaknesses of study, novelty/significance, general execution and scholarship.<br/><br/>

Reviewer #1: In this re-revised manuscript, the authors have meaningfully addressed the points raised in two previous rounds of reviews. No additional comments from me.

Reviewer #3: The authors have now addressed my major concerns regarding the proteomics analysis. They have addressed in the text my concerns about the magnitude of the PTEN or INPP5D phenotypes in their Discussion section.

--------------------

**Part II – Major Issues: Key Experiments Required for Acceptance**

Please use this section to detail the key new experiments or modifications of existing experiments that should be absolutely required to validate study conclusions.<br/><br/>Generally, there should be no more than 3 such required experiments or major modifications for a "Major Revision" recommendation. If more than 3 experiments are necessary to validate the study conclusions, then you are encouraged to recommend "Reject".<br/><br/>

Reviewer #1: No additional comments

Reviewer #3: (No Response)

--------------------

**Part III – Minor Issues: Editorial and Data Presentation Modifications**

Please use this section for editorial suggestions as well as relatively minor modifications of existing data that would enhance clarity.<br/><br/>

Reviewer #1: No additional comments

Reviewer #3: (No Response)

--------------------

PLOS authors have the option to publish the peer review history of their article (what does this mean?). If published, this will include your full peer review and any attached files.

If you choose “no”, your identity will remain anonymous but your review may still be made public.

**Do you want your identity to be public for this peer review?**  For information about this choice, including consent withdrawal, please see our Privacy Policy.

Reviewer #1: No

Reviewer #3: No

# Editor report: Acceptance letter

**DOI:** 10.1371/journal.ppat.1014196.r006

**Authors:** Helena Boshoff

Dear Dr. Russell,

We are delighted to inform you that your manuscript, "Ubiquitylation by the GID/CTLH complex regulates the metabolic and innate immune response of macrophages to infection by Mycobacterium tuberculosis," has been formally accepted for publication in PLOS Pathogens.

We have now passed your article onto the PLOS Production Department who will complete the rest of the pre-publication process. All authors will receive a confirmation email upon publication.

Thank you again for supporting open-access publishing; we are looking forward to publishing your work in PLOS Pathogens.

Best regards,

Dominique Soldati-Favre

Editor-in-Chief

PLOS Pathogens
